# A lymphatic-absorbed multi-targeted kinase inhibitor for myelofibrosis therapy

**DOI:** 10.1038/s41467-022-32486-8

**Published:** 2022-08-17

**Authors:** Brian D. Ross, Youngsoon Jang, Amanda Welton, Christopher A. Bonham, Dilrukshika S. W. Palagama, Kevin Heist, Jagadish Boppisetti, Kasun P. Imaduwage, Tanner Robison, Leah R. King, Edward Z. Zhang, Cyrus Amirfazli, Kathryn E. Luker, Winston Y. Lee, Gary D. Luker, Thomas L. Chenevert, Marcian E. Van Dort

**Affiliations:** 1 Department of Radiology and the Center for Molecular Imaging, University of Michigan School of Medicine, Ann Arbor, MI USA; 2 Department of Biological Chemistry, University of Michigan School of Medicine, Ann Arbor, MI USA; 3 Department of Biomedical Engineering, University of Michigan, Ann Arbor, MI USA; 4 Department of Pathology, University of Michigan School of Medicine, Ann Arbor, MI USA; 5 Department of Microbiology and Immunology, University of Michigan School of Medicine, Ann Arbor, MI USA

**Keywords:** Drug development, Pharmacokinetics, Myeloproliferative disease

## Abstract

Activation of compensatory signaling nodes in cancer often requires combination therapies that are frequently plagued by dose-limiting toxicities. Intestinal lymphatic drug absorption is seldom explored, although reduced toxicity and sustained drug levels would be anticipated to improve systemic bioavailability. A potent orally bioavailable multi-functional kinase inhibitor (LP-182) is described with intrinsic lymphatic partitioning for the combined targeting of phosphoinositide 3-kinase (PI3K) and mitogen-activated protein kinase (MAPK) signaling pathways without observable toxicity. We demonstrate selectivity and therapeutic efficacy through reduction of downstream kinase activation, amelioration of disease phenotypes, and improved survival in animal models of myelofibrosis. Our further characterization of synthetic and physiochemical properties for small molecule lymphatic uptake will support continued advancements in lymphatropic therapy for altering disease trajectories of a myriad of human disease indications.

## Abstract (web summary)

Combination therapies simultaneously inhibiting different therapeutic targets in cancer is challenged by individual pharmacokinetic profiles. Here, the authors generate an orally provided multi-targeted kinase inhibitor that is lymphatic absorbed and increases survival in a murine model of myelofibrosis.

## Introduction

Aberrant signaling pathways in cancer can evade therapy through intrinsic resistance or compensatory mechanisms that drive a resistant state. Activation of oncogenic PI3K and MAPK signaling has inspired development of molecularly targeted drugs, facilitating combination treatment strategies designed to overcome such adaptations due to signaling cross-talk and activation of downstream effectors^1–3^. Nevertheless, treatments using kinase inhibitor combinations remain a challenge clinically as trials have struggled to create positive balance between gains in survival, therapeutic efficacy, and dose-limiting toxicity^4,5^. Single-agent multi-functional kinase inhibitors could deliver consistent and synergistic dosing ratios against multiple targets simultaneously, thereby blocking compensatory oncogenic signaling pathways while minimizing the prospect for adverse effects^6,7^.

Avoiding hepatic first-pass metabolism by sequestration and transport through gastrointestinal lymphatic vessels has potential to improve drug exposure while reducing dose-limiting toxicities often observed in combination therapies^8^. Despite the potential advantages, this route of drug delivery has remained under explored by the pharmaceutical industry likely due in part to limited understanding surrounding the chemical and pharmacokinetic properties required for lymphatic rather than portal venous absorption of small molecules. Prior examples of small molecules demonstrated to achieve lymphatic absorption include vitamins D3 and E, halofantrine and synthetic cannabinoids^9–14^. Although amphiphilic or macromolecular conjugates and controlled release formulations have been evaluated for lymphatic uptake of therapeutic payloads, this strategy results in a majority of the overall molecular composition being therapeutically inert^15–22^. Overcoming the chemical, physical, and biological barriers of small molecule lymphatic absorption will lessen the paucity of future lymphatically-directed drug discovery efforts.

Using synthetic medicinal chemistry along with computational docking studies, we report the development of a potent and selective, orally bioavailable, single-molecule multi-functional kinase inhibitor (LP-182) against PI3K and MAPK signaling pathways. The unique physiochemical qualities of LP-182 deviate from prior studies in that the bulk chemical structure is therapeutically active, achieving lymphatic absorption following oral administration. Sequestration of LP-182 in the mesenteric lymphatic system results in extended drug and active metabolite levels in systemic blood circulation, improving overall bioavailability and diminishing observable toxicity. Pre-clinical efficacy of LP-182 in the myeloproliferative leukemia oncogene (*MPL*^*W515L*^) mouse model of myelofibrosis (MF)^23^, a hematologic neoplasm with marked involvement of secondary lymphoid organs such as the spleen^24^, was demonstrated by amelioration of splenomegaly and fibrotic disease phenotypes, and extended overall survival. Myelofibrosis arises predominantly from mutations in hematopoietic stem and progenitor cells (HSPCs) that activate Janus kinase 2 (JAK2), and resistance to standard-of-care JAK2 inhibitors (Ruxolitinib, Fedratinib) has been shown as a consequence of concomitant activation of compensatory PI3K and MAPK survival signaling^25–27^. Oral treatment of *MPL*^*W515L*^ mice with LP-182 also resulted in renormalization of blood cell populations and attenuation of downstream protein kinase B (PKB/AKT) and extracellular signal-regulated protein kinase 1/2 (ERK1/2) signaling activity in bone marrow and isolated splenocytes. Further demonstration of a synthetic scheme for generating classes of lymphatic multi-targeted kinase inhibitors, and characterization of physiochemical features central to lymphatic uptake, provides a path to advance the application of small molecule lymphatropic therapies in treatment of human disease.

## Results

### Design and activity of the multi-functional kinase inhibitor LP-182

**Fig. 1:**
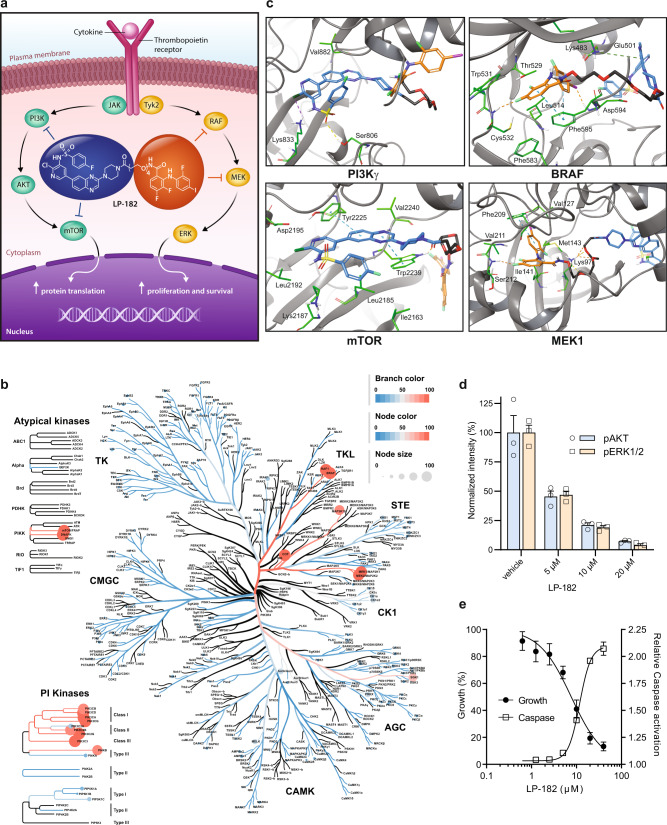
Multi-functional kinase inhibitor LP-182 exhibits selectivity and specificity. **a** Targeting of downstream PI3K/mTOR and RAF/MEK signaling nodes by LP-182 in hematopoietic cells with constitutive JAK activation. **b** Single-point broad panel kinome screening against 2.5 μM LP-182. Average percent kinase inhibition from replicate data was input into Coral Human Kinome Visualization software, and analyzed as described in Methods^61–63^. Scaling for branch color, node color, and node size represents percent inhibition as indicated. Source data are provided as a Source Data file. Kinase families: Tyrosine kinase, TK; Tyrosine kinase-like, TKL; Serine/threonine kinase, STE; Casein kinase 1, CK1; Protein kinase A/G/C, AGC; Ca^2+^/calmodulin-dependent protein kinase, CAMK; CDK/MAPK/GSK/CDK-like, CMGC; Phosphoinositol kinases, PI Kinases. **c** Docked structures of LP-182 at the PI3Kγ (PDB code 3L08), mTOR1 (PDB code 4JSX), and BRAF (PDB code 5HI2) catalytic sites, and MEK1 (PDB code 3ORN) allosteric pocket as indicated^28, 31–33^. Molecular notation shows PI3K/mTOR inhibitor of LP-182 in blue, RAF/MEK inhibitor in orange, and polyethylene glycol linker in black. **d** Normalized fluorescence intensity values of phosphorylated AKT (pAKT; pS473) and ERK1/2 (pERK1/2; pT202/pY204) flow cytometry staining in SET-2 cells following treatment with indicated concentrations of LP-182 for 16 h. **e** Growth inhibition and Caspase activation values from SET-2 cells treated with indicated concentrations of LP-182 for 72 h or 48 h, respectively. Data represent the mean ± s.e.m., *n* = 3 independent experiments performed in replicate. Corrected data were normalized to vehicle treated control values and analyzed by non-linear regression where indicated. Source data are provided as a Source Data file.

To develop LP-182 as a single small molecule inhibitor of PI3K, mechanistic target of rapamycin (mTOR), and mitogen-activated protein kinase kinase (MEK), we synthesized a novel pan-PI3K and mTOR inhibitor, LP-527 (**7**; a modified analog of GSK2126458^28^), to facilitate its chemical attachment to the MEK inhibitor analog PD0316684 (a close structural analog of PD0325901^29^) via a short polyethylene glycol (PEG) linker (Fig. 1a, Supplementary Figs. 1 and 2). Selectivity of LP-182 was maintained when tested against a broad panel of 321 human protein kinases at ≥100-fold IC_50_ of either single agent, targeting specifically PI3K and MAPK signaling nodes (Fig. 1b, Supplementary Fig. 3). In addition to PI3K, mTOR, and MEK, we observed potent inhibition of rapidly accelerated fibrosarcoma (RAF) kinases, demonstrating LP-182 as a multi-functional kinase inhibitor against high value therapeutic targets in human disease (Fig. 1a, b, Supplementary Fig. 3). Docking analysis in the ATP-binding site of PI3Kγ displayed hydrogen-bonding interactions for Lys833 with the sulfonamide nitrogen, Ser806 with the sulfonyl oxygen, and Val882 with the quinoxaline nitrogen of LP-182, while the fluorophenyl group rested in a hydrophobic region in the back pocket of the enzyme, all similar to that observed for the structural analog GSK2126458^28^ (Fig. 1c, Supplementary Fig. 4a, b). In the ATP-binding pocket of mTOR, potential π-stacking between Tyr2225 and Trp2239 with the benzyl and quinoxaline rings of LP-182 was observed, while the fluorophenyl group also rested in a hydrophobic pocket in the N-lobe. Additionally, hydrogen-bonding between Glu2190 and the sulfonamide nitrogen was displayed, although positioning of Asp2195 may also support potential hydrogen-bonding here, and of Val2240 with the quinoxaline nitrogen (Fig. 1c, Supplementary Fig. 4c, d). Modeling of BRAF exhibited strong contributions from both aromatic A and B rings of LP-182. Evidence for π-stacking of both rings with Phe595 was observed, as well as electrostatic interactions between the B ring 4-iodine atom and the carbonyl oxygen of Asp594 within the DFG motif, while the A ring difluorophenyl sat in the hydrophobic nucleotide-binding pocket with the 4-fluorine atom making hydrogen-bonding contacts with the carbonyl oxygen of Cys532 (Fig. 1c, Supplementary Fig. 5a, b). Docking of LP-182 in the MEK1 allosteric binding pocket suggested the presence of hydrogen-bonding interactions between the hydroxamate and ethylene glycol oxygen atoms of the linker with Lys97 and between the 4-fluorine atom of the aromatic A ring and the amide backbone of Ser212. Presence of π-stacking between the aromatic B ring and Phe209, as well as hydrogen-bonding interactions between the iodine atom and carbonyl oxygen of Val127 of the hinge region were also observed, and together are consistent with the structurally analogous MEK inhibitors PD0325901 and CH4987655^30,31^ (Fig. 1c, Supplementary Fig. 5c, d). Treatment of the human megakaryoblastic cell line SET-2 (harboring the JAK2 V617F driver mutation of MF) with LP-182 decreased downstream activation of both AKT and ERK1/2 by ~50% at 5 μM, inhibited cellular proliferation with an IC_50_ of 7.6 ± 0.82 μM, and initiated apoptotic cell death (Fig. 1d, e). These data suggest LP-182 retains multi-functional inhibition profiles and mechanisms of action against target kinases through binding interactions similar to those of the single agent inhibitor analogs^28,30–33^.

### Sustained oral bioavailability of LP-182 and metabolite inhibitors

**Fig. 2:**
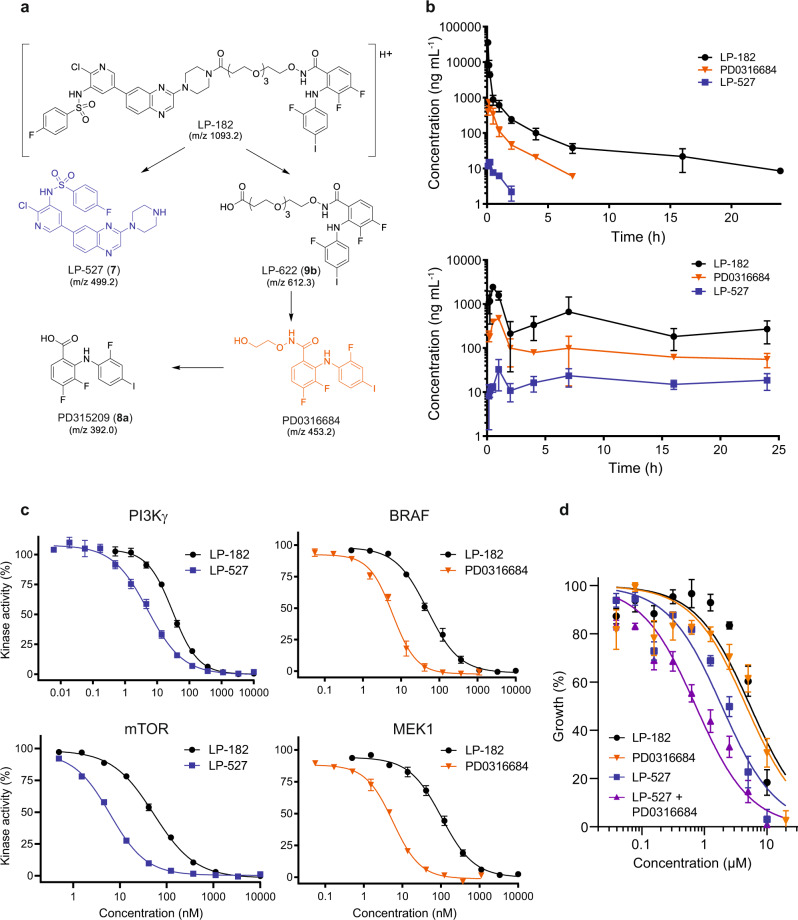
In vivo pharmacokinetics upon oral administration achieves sustained levels of LP-182 and potent biologically active metabolites. **a** LP-182 in vivo metabolic degradation products. **b** In vivo pharmacokinetics of LP-182 and associated metabolites in mice following a single intravenous (15 mg kg^−1^; top panel) or oral (400 mg kg^−1^; bottom panel) dose of LP-182. Data represent the mean ± s.d., *n* = 3 animals sampled per time point per compound dosing. Source data are provided as a Source Data file. **c** Target kinase (PI3Kγ, mTOR, BRAF, MEK1) 10-point inhibition assays against LP-182, LP-527, or PD0316684 at the indicated concentrations. Data represent the mean ± s.e.m., LP-182 (*n* = 5, PI3Kγ; *n* = 3, mTOR; *n* = 3, BRAF; *n* = 5, MEK1; independent experiments performed in replicate), LP-527 (*n* = 5, PI3Kγ; *n* = 3, mTOR; independent experiments performed in replicate), PD0316684 (*n* = 1, BRAF; *n* = 1, MEK1; independent experiments performed in replicate). Calculated IC_50_ values from replicate data are supplied in Supplementary Fig. 7. Source data are provided as a Source Data file. **d** Growth inhibition values from SET-2 cells treated with indicated concentrations of LP-182, PD0316684, LP-527, or LP-527 and PD0316684 for 72 h. Data represent the mean ± s.e.m., *n* = 3 independent experiments performed in replicate. Corrected data were normalized to vehicle treated control values and analyzed by non-linear regression where indicated. Source data are provided as a Source Data file.

The in vivo disposition of LP-182 was evaluated to define its pharmacological activity following intravenous (15 mg kg^−1^) and oral (400 mg kg^−1^) administration in mice. Pharmacokinetic analysis of blood concentrations over a 24 h period identified that degradation profiles of LP-182 occurred through decoupling of individual PI3K/mTOR and RAF/MEK inhibitors to the active metabolites LP-527 and PD0316684, respectively (Fig. 2a). Oral administration of LP-182 exhibited prolonged systemic circulation for the parent molecule and all active metabolites as compared to intravenous, with single agent metabolites LP-527 and PD0316684 maintaining levels of approximately 20 and 100 ng mL^-1^ over 24 h, respectively (Fig. 2b, Supplementary Fig. 6a). Plasma half-life (t_1/2_) of LP-182 was 12.7 h for oral versus 7.9 h for intravenous, and sustained levels of ~300 ng mL^−1^ were also observed over a 24 h period after oral dosing (Fig. 2b). Quantitative determination of LP-182 stability in isolated murine liver microsomes revealed a t_1/2_ of ~8 min suggesting that hepatic metabolism may play a central role in regulating systemic levels of LP-182 and its metabolite inhibitors (Supplementary Fig. 6b). Dose range studies over 72 h revealed the majority of orally administered LP-182 was recovered from fecal samples collected after 24 and 48 h (~95% and 5% of total recovered respectively), with minimal compound detected after 72 h, and negligible amounts in the urine across all time points (Supplementary Fig. 6c). Dose-dependent recovery of LP-182 in feces correlated linearly with oral dose delivered (R^2^ = 0.9834), showing an ~20% dose-independent fecal excretion (% drug recovered) of intact LP-182, setting a lower threshold limit of ~80% oral bioavailability (Supplementary Fig. 6c, d). While in vivo metabolic decomposition of LP-182 occurred, individual metabolites are highly active with ~5–10 fold greater potency (0.6 nM ≤ IC_50_ ≤ 18 nM) than the parent inhibitor LP-182 (2 nM ≤ IC_50_ ≤ 350 nM) across all targets in vitro (Fig. 2c, Supplementary Fig. 7). However, LP-182 levels remained ~3- to 10-fold higher than that of LP-527 and PD0316684 upon oral administration suggesting the potential for a collective contribution to therapeutic efficacy from both LP-182 and the individual metabolite inhibitors (Fig. 2b). Treatment of SET-2 cells with the active metabolites LP-527 and PD0316684 in combination also led to >5-fold increase in inhibition of cellular proliferation (IC_50_ 0.73 ± 0.071 µM) as compared to treatment with LP-182, or >2-fold increase as compared to each individual metabolite in isolation (Fig. 2d). Thus, the balance among potencies, plasma concentrations, and prolonged systemic oral bioavailability of LP-182 and its metabolites may contribute to reduced toxicity and an overall net therapeutic benefit in vivo.

### Lymphatic absorption of LP-182

**Fig. 3:**
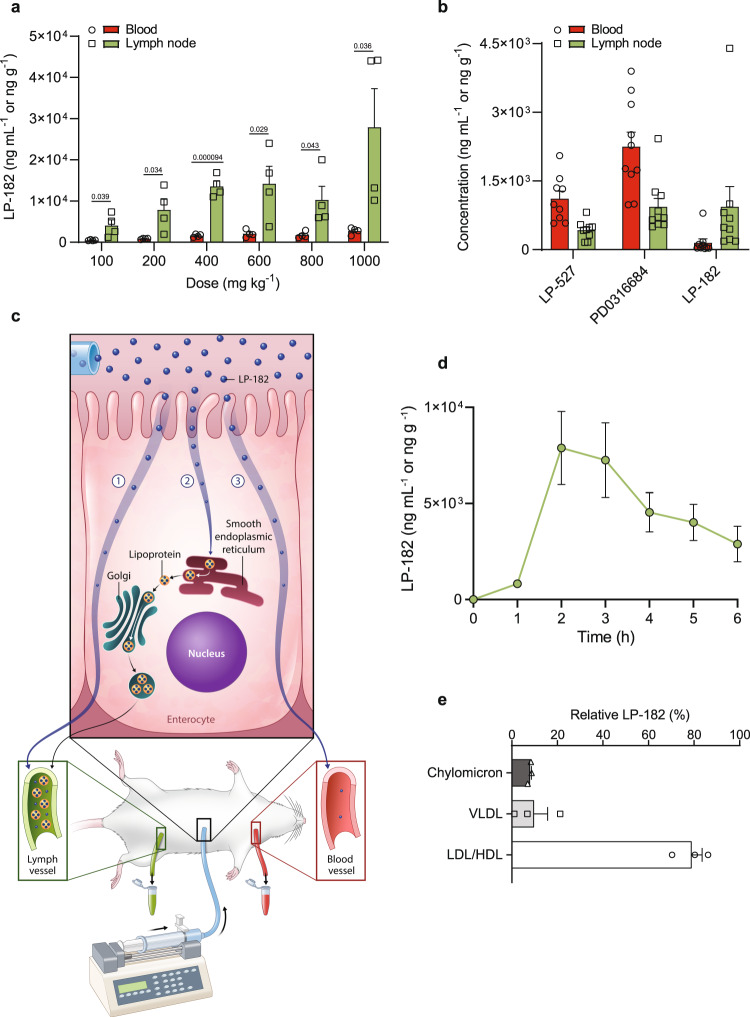
Absorptive lymphatic partitioning of LP-182 involves association with lipoproteins. **a** Concentration of LP-182 in blood and mesenteric lymph nodes from mice 30 min following oral administration at the indicated doses. Data represent the mean ± s.e.m., *n* = 4 animals per dose. Statistical significance determined using multiple unpaired t-test corrected with Holm-Šidák multiple comparisons test (100 mg kg^−1^, *p* = 0.039; 200 mg kg^−1^, *p* = 0.034; 400 mg kg^−1^, *p* = 0.000094; 600 mg kg^−1^, *p* = 0.029; 800 mg kg^−1^, *p* = 0.043; 1000 mg kg^−1^, *p* = 0.036). **b** Concentration of LP-527, PD0316684, and LP-182 in blood and mesenteric lymph nodes from mice 4 h following oral administration at 100 mg kg^−1^. Data represent the mean ± s.e.m., LP-527 (*n* = 9), PD0316684 (*n* = 10), and LP-182 (*n* = 9) animals per group. **c** Enterocyte schematic showing possible routes of drug access into the blood and intestinal lymphatics in an anesthetized rat mesenteric lymph cannulation model^8,37^. **d** Kinetic profiles of LP-182 in lymphatic fluid from anesthetized rats following 1 h intra-duodenal infusion at 50 mg kg^−1^. Data represent the mean ± s.e.m., *n* = 7 animals per time point. Source data are provided as a Source Data file. **e** Relative percent of total LP-182 measured within chylomicron, very low-density lipoprotein (VLDL), and low-density lipoprotein/high-density lipoprotein (LDL/HDL) fractions upon separation of pooled time course lymphatic fluid by ultracentrifugation from anesthetized rats following 1 h intra-duodenal infusion of LP-182 at 50 mg kg^−1^. Data represent the mean ± s.e.m., *n* = 3 animals.

Chemical properties such as good water solubility (calculated partition coefficient; clogP <5) and small molecular weight (M.W. < 500 Da) facilitate oral drug absorption from the interstitial space via blood capillaries^34^. Prolonged systemic stability, circulation, and combined physiochemical properties contradict this mode of oral absorption for LP-182, leading to our consideration that intestinal lymphatic absorption may occur rather than a direct hematogenous route. Evaluation of mouse blood and mesenteric lymph nodes at peak blood levels (30 min) showed a dose-dependent increase of LP-182 (5-15 μM) in lymph nodes following oral administration, with an ~8:1 lymph/plasma ratio that remained relatively constant over the dose range (Fig. 3a). Oral administration of individual metabolites LP-527 and PD0316684 exhibited reduced lymphatic uptake (0.4:1 and 0.5:1 lymph/plasma, respectively) in the mesenteric lymph nodes 4 h post-administration as compared to LP-182 administered at ~0.5 molar equivalents (each compound dosed at 100 mg kg^-1^), revealing selectivity and increased levels of LP-182 lymphatic absorption (~8:1 lymph/plasma) (Fig. 3b). Most drugs undergo first-pass drug metabolism in the liver upon oral administration which limits exposure to the systemic circulation and may cause hepatic injury. Re-routing drug absorption from portal venous blood to intestinal lymphatics has been shown to improve oral bioavailability and reduce off-target toxicity^35,36^. Healthy mice treated orally with vehicle or LP-182 (400 mg kg^−1^) for 10 d followed by 8 d rest exhibited no adverse effects, change in body weight, or change in complete blood counts. Furthermore, necropsy revealed no histopathological abnormalities, indicating LP-182 is well tolerated with no observed toxicity (Supplementary Fig. 8). Thus, lymphatic uptake of LP-182 may provide a reservoir for gradual release into the systemic circulation, minimizing toxicity by controlling plasma levels and exposure duration of LP-182 and its metabolites to maintain a persistent therapeutic dosage.

Mechanisms of drug entry into lymphatics are varied and complex. To examine further the lymphatic absorption of LP-182, we employed an anesthetized rat mesenteric lymph duct cannulation model^37^. Lipophilic compounds may be absorbed across intestinal enterocytes directly into the lymphatics (Fig. 3c; ➀) or may enter synthetic lipoprotein assembly pathways through binding affinity to the interfacial region of chylomicrons, very low-density lipoproteins (VLDLs), and high-density lipoproteins (HDLs)^38–41^. Following enterocyte exocytosis, drug–lipoprotein complexes are then transported across the basement membrane and traffic into lymphatics (Fig. 3c; ➁). Alternatively, compounds may be absorbed across the enterocyte into vascular capillaries (Fig. 3c; ➂)^8^. Consistent with results obtained from mouse mesenteric lymph node studies, kinetic profiling of lymphatic fluid over a 6 h period revealed almost exclusive intestinal lymphatic uptake of LP-182 (Fig. 3d), while levels in the blood were negligible and found to account for only 1.7% ± 0.4 of total compound measured among each set of temporal fractions, on average. Fractionation of lymphatic fluid by ultracentrifugation further revealed association of LP-182 with chylomicron (8.0% of total), VLDL (9.7% of total), and HDL (79.0% of total) fractions (Fig. 3e). Together, these findings provide evidence that selective LP-182 lymphatic entry and uptake may occur through association with lipoprotein fractions within intestinal enterocytes upon oral administration. Subsequent processing and transport into the lymphatic system would then promote the prolonged systemic bioavailability and reduced toxicity profiles observed, as compared to individual small molecule agents alone.

### LP-182 ameliorates myelofibrosis phenotypes in vivo

**Fig. 4:**
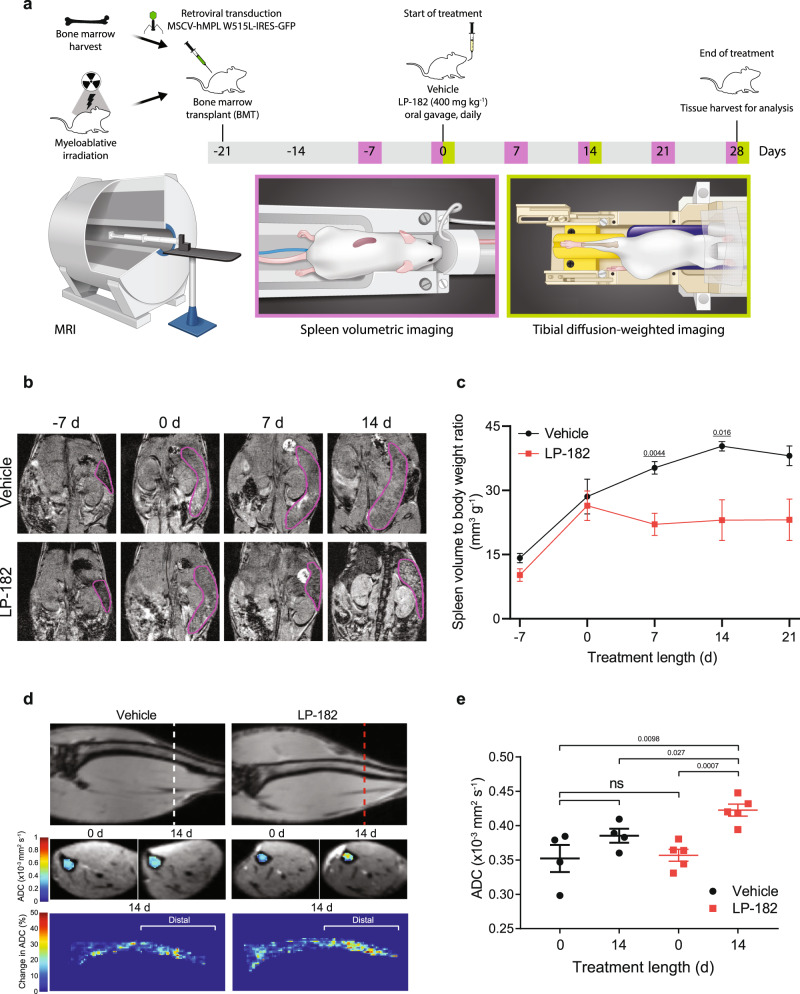
Magnetic resonance imaging of LP-182 treatment response reveals reduced splenomegaly and tibia bone marrow cellularity in the *MPL*^*W515L*^ mouse model of myelofibrosis. **a** Schematic for *MPL*^*W515L*^ myelofibrosis mouse model experimental design and daily oral treatment regimen with vehicle or LP-182 (400 mg kg^−1^). **b** Representative coronal MR images highlighting spleen cross sections (purple) on the indicated day of treatment with vehicle or LP-182. **c** Spleen volume to body weight measurements on the indicated day of treatment with vehicle or LP-182. Data represent the mean ± s.e.m., vehicle (*n* = 2, day 21 timepoint; *n* = 4, all other timepoints), LP-182 (*n* = 5) animals per group. Source data are provided as a Source Data file. Statistical significance determined using two-tailed unpaired *t*-test (7 d, *p* = 0.0044; 14 d, *p* = 0.016). **d** Coronal and axial MR images through the tibia showing apparent diffusion coefficient (ADC) values as pseudocolored heat-maps (middle panels) overlaid on distal axial slices (hashed lines; top panels) at the indicated day of treatment with vehicle or LP-182. Tibia were manually segmented and ADC values within the bone marrow space averaged along lines perpendicular to the coronal plane yielding a two-dimensional perspective of percent ADC change relative to 0 d (color heat-maps; bottom panels). **e** Distal tibia bone marrow multi-slice average ADC measurements at the indicated day of treatment with vehicle or LP-182. Data represent the mean ± s.e.m., vehicle (n = 4), LP-182 (n = 5) animals per group. Statistical significance determined using two-tailed unpaired *t*-test (Vehicle 0 d vs. Vehicle 14 d, *p* = 0.19; Vehicle 0 d vs. LP-182 0 d, *p* = 0.82; Vehicle 0 d vs. LP-182 14 d, *p* = 0.0098; Vehicle 14 d vs. LP-182 14 d, *p* = 0.027; LP-182 0 d vs. LP-182 14 d, *p* = 0.0007).

Myelofibrosis is a myeloproliferative neoplasm (MPN) caused primarily by mutations that activate JAK2 signaling, resulting in splenomegaly and a marked expansion of hematopoietic cells in bone marrow^24,42^. Given the pronounced involvement of secondary lymphoid tissues in MF, we hypothesized that LP-182 would be effective against key manifestations of disease in a mouse model driven by an activating mutation in the thrombopoietin receptor (*MPL*^*W515L*^)^23^. Daily oral treatment with LP-182 (400 mg kg^−1^) or vehicle began 21 d after transplantation of *MPL*^*W515L*^ transduced HSPCs into lethally irradiated recipient mice, when spleen volumes measured by magnetic resonance imaging (MRI) exceeded wild-type by ~5-10 fold. Volumetric MRI was used to measure spleen volume every 7 d and diffusion-weighted MRI used to monitor cellular density within the tibial bone marrow space as an apparent diffusion coefficient (ADC) every 14 d after treatment initiation^43^. Mice were euthanized after 28 d treatment or upon approaching a moribund state, and tissues were harvested for analysis (Fig. 4a). LP-182 treatment significantly stabilized spleen volume measurements after 7 d and prevented further progression of splenomegaly over 21 d, resulting in an ~35% reduction after 14 d as compared to vehicle which reached 40.3 ± 1.1mm^3^g^−1^, similar to previous reports (Fig. 4b, c)^27^. Anatomical tibia MR images overlaid with corresponding axial and coronal pseudocolor ADC heat-maps illustrated a spatial heterogeneity of ADC change throughout the entire bone marrow. Treatment with LP-182 resulted in a variable, spatially-dependent increase in ADC values, with the majority of ADC change occurring in the distal half of the tibia indicative of a loss in cell density within the marrow space upon treatment (Fig. 4d, e).

**Fig. 5:**
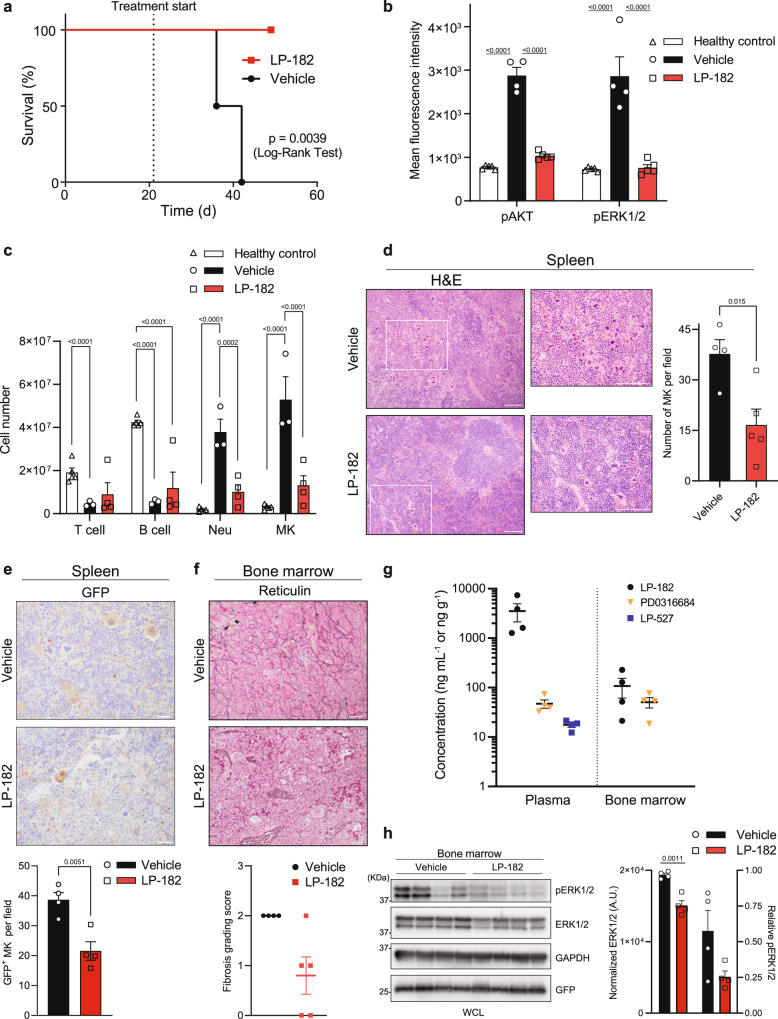
LP-182 attenuates signal transduction and restores immune cell balance to improve cellular disease phenotypes and survival in the *MPL*^*W515L*^ MF mouse model. **a** Kaplan–Meier plot of MF mice treated as indicated. Data represent vehicle (*n* = 4), LP-182 (*n* = 5) animals per group. Statistical significance determined using Log-Rank (Mantel-Cox) test**. b** Mean fluorescence intensity values of pAKT (pS473) and pERK1/2 (pT202/pY204) in splenocytes from healthy control and MF mice treated as indicated. Data represent the mean ± s.e.m., healthy control (*n* = 5), vehicle (*n* = 4), LP-182 (*n* = 5) animals per group. Statistical significance determined using Two-way ANOVA corrected with Tukey’s multiple comparisons test (pAKT Healthy control vs. Vehicle, *p* < 0.0001; pAKT Vehicle vs. LP-182, *p* < 0.0001; pERK1/2 Healthy control vs. Vehicle, *p* < 0.0001; pERK1/2 Vehicle vs. LP-182, *p* < 0.0001). **c** Immune cell numbers from spleen of healthy **c**ontrol and MF mice treated as indicated. Data represent the mean ± s.e.m., healthy control (*n* = 5), vehicle (*n* = 3), LP-182 (*n* = 4) animals per group. Statistical significance determined using Two-way ANOVA corrected with Tukey’s multiple comparisons test (T cell Healthy control vs. Vehicle, *p* = 0.049; B cell Healthy control vs Vehicle, *p* < 0.0001; B cell Healthy control vs. LP-182, *p* < 0.0001; Neu Healthy control vs. Vehicle, *p* < 0.0001; Neu Vehicle vs. LP-182, *p* = 0.0002; MK Healthy control vs. Vehicle, p < 0.0001; MK Vehicle vs. LP-182, p < 0.0001). **d** Representative histological images and quantitation of MK cells per high-power field from spleen of MF mice treated as indicated. Hematoxylin & Eosin (H&E; 10x with 20x inset, scale 100 µm**)**. Data represent the mean ± s.e.m., vehicle (*n* = 4), LP-182 (*n* = 5) animals per group. Statistical significance determined using two-tailed unpaired *t*-test (*p* = 0.015). **e** Representative immunohistochemistry images with quantitation of GFP-positive MK cells per high-pow**e**r field from spleen of MF mice treated as indicated. Green Fluorescent Protein (GFP; 40x, scale 20 µm). Data represent the mean ± s.e.m., vehicle (*n* = 4), LP-182 (*n* = 4) animals per group. Statistical significance determined using two-tailed unpaired *t*-test (*p* = 0.0051). **f** Representative histological images with scored fibrosis grading from bone marrow of MF mice treated as indicated. Reticulin (40x, scale 20 µm). Data represent the mean ± s.e.m., vehicle (*n* = 4), LP-182 (*n* = 5) animals per group. Individual data sets and scoring details are provided in Supplementary Table 1. **g** Concentration of LP-182 and associated metabolites in serum and bone marrow from MF mice ~2 h following final oral administration. Data represent the mean ± s.e.m., *n* = 4 animals. **h** Immunoblot analysis and quantitation of ERK1/2 and pERK1/2 (pT202/pY204) from bone marrow of MF mice treated as indicated. Data were normalized to GAPDH and analyzed relative to ERK1/2 where indicated. Data represent the mean ± s.e.m., vehicle (*n* = 4), LP-182 (*n* = 4) animals per group. Statistical significance determined using two-tailed unpaired *t*-test (*p* = 0.0011). Source data are provided as a Source Data file. a-h *MPL*^*W515L*^ MF mice were treated p.o. daily with vehicle (14-21 d) or LP-182 (28 d) at 400 mg kg^−1^.

All mice treated with LP-182 survived 50 d post-transplant (28 d treatment), the planned end of the study, whereas vehicle-treated mice showed progressive disease, reaching humane endpoints that limited statistical comparisons between groups to <21 d. One mouse in the vehicle group died from procedural complications, not progressive MF, and was excluded entirely from all analyses. Kaplan-Meier curve revealed that LP-182 significantly improved overall survival in *MPL*^*W515L*^ mutant MF mice (Fig. 5a). Further evaluation of disease progression by flow cytometry, histology, LCMS/MS, and immuno-blotting revealed on-target inhibition by LP-182 and amelioration of MF phenotypes in vivo. Splenocytes from mice treated with vehicle for 14–21 d showed markedly elevated levels of AKT and ERK1/2 activation as quantified by intracellular staining for AKT and ERK1/2 phosphorylation (Fig. 5b). By comparison, treatment with LP-182 for 28 d reduced active AKT and ERK1/2 levels to those comparable to healthy control mice, suggesting that LP-182 and/or metabolite inhibitors localize to the spleen and block downstream *MPL*^*W515L*^ signal transduction through PI3K and MAPK pathways (Fig. 5b). LP-182 treatment also decreased cell numbers of neutrophils (Neu) and megakaryocytes (MK) in the spleen to levels resembling those of healthy mice, thus normalizing two major cell lineages driving pathogenesis in MF (Fig. 5c, Supplementary Fig. 9)^44^. Histological tissue sections from mice treated with vehicle for 14–21 d showed morphologic hallmarks of MF, including prominent megakaryocytic hyperplasia with atypical morphology and increased reticulin fibrosis. These mice also develop marked extramedullary hematopoiesis (with a predominance of maturing megakaryocytic and granulocytic elements) in the liver and spleen, resulting in the effacement of normal tissue architectures. Treatment with LP-182 for 28 d displayed less severe pathologic changes in splenic pulp structures and bone marrow, showing reduced extramedullary hematopoiesis, number of MKs and *MPL*^*W515L*^-positive MKs (green fluorescent protein staining; GFP), and diminution of reticulin fibrosis (Fig. 5d–f, Supplementary Fig. 10a, b, Supplementary Table 1). Further analysis of blood plasma from MF mice ~2 h after final treatment found LP-182, LP-527, and PD0316684 levels similar to those observed in healthy mice, while analysis of bone marrow revealed that LP-182 and active metabolites can also localize to this primary region of HSPCs (Fig. 2b, Fig. 5g). Bone marrow from mice treated with vehicle for 14 – 21 d showed elevated levels and activation of ERK1/2 as compared to mice treated with LP-182 for 28 d, suggesting that distribution of LP-182 and metabolite inhibitors to the bone marrow can attenuate downstream *MPL*^*W515L*^ signal transduction (Fig. 5h). These results underpin the therapeutic benefit provided by LP-182 treatment as observed by quantitative differences in spleen volume and changes in cellularity between vehicle and treated groups by MRI (Fig. 4).

In an additional set of studies, we sought to assess whether the effects on disease progression may occur through direct targeting of cells that possess constitutive thrombopoietin receptor activation. Here, daily oral treatment with LP-182 (400 mg kg^−1^) or vehicle began 15 - 18 d after transplantation and was set for 14 d due to the aggressive disease phenotypes observed in untreated animals, with only spleen volume measurements being recorded by MRI every 7 d after treatment initiation (Study 2). Although less pronounced, the rate of progressive splenomegaly in vehicle treated animals closely matched that of the previous cohort, and consistent with this, LP-182 significantly attenuated spleen volume increase by ~35% after 14 d treatment compared to vehicle (Supplementary Fig. 11a). Comparison of spleen volume to spleen weight among the separate studies revealed an average of 1.04 ± 0.06 and 1.07 ± 0.05 mm^3^ mg^−1^, respectively, validating the accuracy and consistency of in vivo MRI measurements for estimating spleen size or mass (Supplementary Fig. 11b). Evaluating the presence of HSPC-derived cells transduced with *MPL*^*W515L*^ via GFP expression showed that GFP-positive *MPL*^*W515L*^ neutrophil frequency was reduced in LP-182 treated mice, and this was associated with reductions in GFP-positive granulocyte-monocyte progenitor (GMP) frequency, and myeloid progenitor (MP) and megakaryocyte-erythrocyte progenitor (MEP) frequencies (Supplementary Fig. 11c–e). Overall, these data suggest that in vivo bioavailability and distribution of LP-182 and/or its metabolite inhibitors to secondary lymphoid tissues attenuates PI3K and MAPK signaling downstream of *MPL*^*W515L*^ to alleviate constitutional and cellular disease phenotypes, renormalize targeted cell populations, and improve overall survival in animal models of MF.

### Development of lymphatropic multi-functional kinase inhibitors

**Fig. 6:**
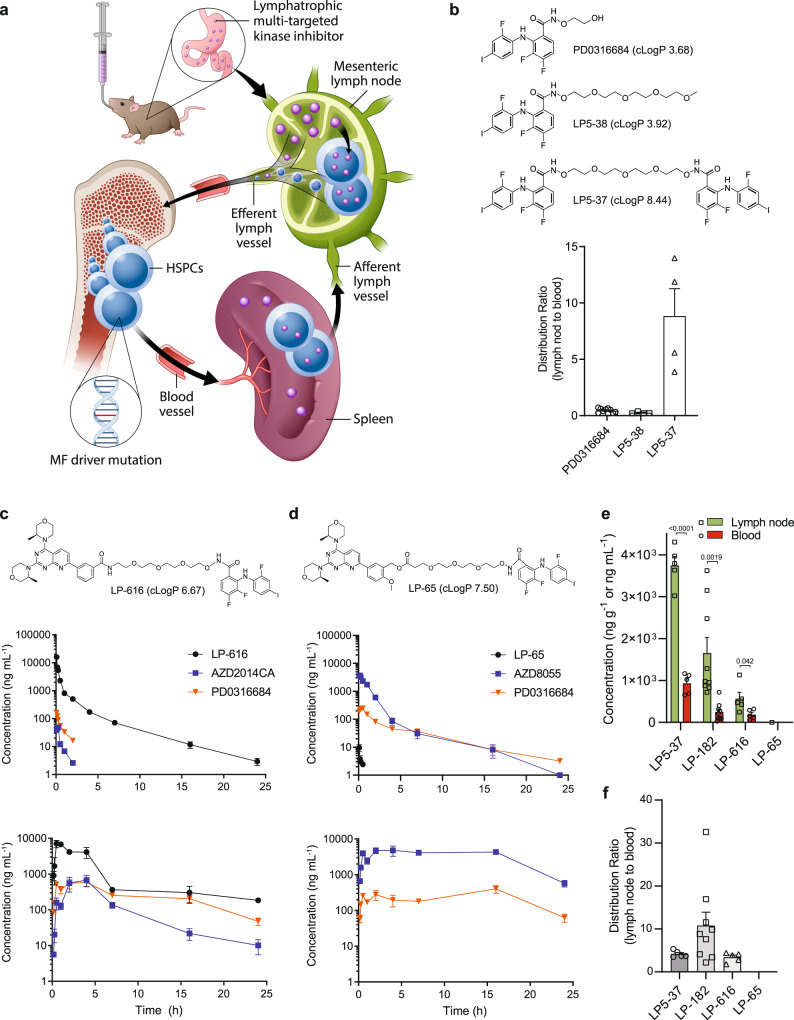
Physiochemical properties influence lymphatic uptake and release of lymphatropic kinase inhibitors into systemic circulation. **a** Schematic model of the lymphatic absorption process for multi-targeted kinase inhibitors following oral administration. Prolonged drug release from mesenteric lymph into the systemic circulation supports sustained plasma levels to diminish compensatory and resistance mechanisms in human diseases such as myelofibrosis (MF). **b** Mesenteric lymph node to blood ratio of PD0316684, LP5-38, and LP5-37 in mice 4 h following oral administration at 100 mg kg^−1^. Data represent the mean ± s.e.m., LP5-38 (*n* = 4), LP5-37 (*n* = 4), PD0316684 (*n* = 10; Fig. 3b) animals per group. In vivo pharmacokinetics of **c** LP-616 or **d** LP-65 and associated metabolites in mice following a single intravenous (15 mg kg^−1^; top panel) or oral (200 mg kg^−1^; bottom panel) dose of LP-616 or LP-65. Data represent the mean ± s.e.m., LP-616 (*n* = 6, 10 and 60 min timepoints, oral; *n* = 3, all other timepoints, intravenous and oral), LP-65 (*n* = 3, all timepoints, intravenous and oral) animals per group. Source data are provided as a Source Data file. **e** Concentration and **f** mesenteric lymph node to blood ratio of LP5-37, LP-182, LP-616, and LP-65 in mice 4 h following oral administration at 400 mg kg^−1^. Data represent the mean ± s.e.m., LP5-37, LP-616, and LP-65 (*n* = 5), LP-182 (*n* = 9) animals per group. Statistical significance determined using two-tailed unpaired *t*-test (LP5-37, *p* < 0.0001; LP-182, *p* = 0.0019; LP-616, *p* = 0.042).

Unique physiochemical properties of LP-182, including a clogP of 7.6 and chemical design utilizing a hydrophilic linker coupled to relatively hydrophobic pharmacophores, presents an important therapeutic paradigm for development of orally bioavailable drugs capable of intestinal lymphatic uptake without nanostructure packaging or attachment to a bulky carrier molecule. LP-182 demonstrates fundamental viability of lymphatropic oral agents, providing the synthetic groundwork to explore additional chemical designs for broadening multi-targeted inhibitor combinations and characterization of the features central to small molecule lymphatic uptake (Fig. 6a). To investigate the impact of lipophilicity on intestinal lymphatic absorption, we generated a compound series (LP5-38 & LP5-37) through chemical attachment of a short PEG moiety to the MEK inhibitor analog PD0316684 that achieved a range of cLogP values from ~3.7 to 8.4 (Fig. 6b). Oral dosing of PD0316684 and LP5-38 (cLogP ~3.8) displayed reduced uptake in the mesenteric lymph nodes 4 h post-administration as compared to ~0.5 molar equivalents of LP5-37 (cLogP ~8.4; each dosed at 100 mg kg^−1^), suggesting a possible relationship between elevated cLogP through linker conjugation and absorptive lymphatic uptake upon oral administration in mice (Fig. 6b). Subsequent design and synthesis of dual mTOR/MEK inhibitors LP-65 and LP-616 through coupling of AZD8055^45^ or its acid *α’*-demethoxy analog AZD2014 (AZD2014CA)^46^ with PD0316684 enabled us to evaluate collectively the impact of cLogP, PEG linker stability, and substitution of chemical inhibitor entities on lymphatic uptake and oral bioavailability. Pharmacokinetic analysis following intravenous (15 mg kg^−1^) and oral (200 mg kg^−1^) administration of LP-616 and LP-65 (cLogP 6.7 and 7.5, respectively) in mice identified similar metabolic decoupling of parent inhibitors to active metabolites AZD2014CA, AZD8055, and PD0316684 as observed for LP-182 (Fig. 6c, d, Supplementary Fig. 12). Systemic circulation of intravenous and orally administered LP-616 closely mirrored that of LP-182 with extended oral bioavailability of both the parent molecule and active metabolites (Fig. 6c, Supplementary Fig. 12a). Insertion of an ester linkage by design resulted in rapid metabolic decomposition of the parent molecule LP-65 to undetectable levels within 1 h of administration, however, systemic circulation of active metabolites also closely mirrored those of LP-182 with extended oral bioavailability (Fig. 6d, Supplementary Fig. 12b). In direct comparison, mesenteric lymph node levels of LP5-37 and LP-616 were significantly higher than in blood 4 h after oral administration at 400 mg kg^−1^, similar to that observed for LP-182, while LP-65 levels remained undetectable among both matrices (Fig. 6e). Consistent with evaluation of LP5-37 and LP5-38, lymphatic uptake also increased with increasing cLogP (LP5-37 > LP-182 > LP-616), however the lymph node to blood ratio was most pronounced for LP-182 suggesting that chemical tuning of cLogP may provide opportunities to control directly lymphatic absorption and/or partitioning (Fig. 6f). In contrast, LP-65 active metabolite and short-lived precursor levels (AZD8055 & LP-622) were elevated in both blood and lymph node as compared to LP-616 and LP-182 metabolic equivalents (AZD2014CA & LP6-26 and LP-527 & LP-622, respectively) (Supplementary Fig. 12c). Together, these data suggest that both LP-65 and LP-616 undergo lymphatic uptake, and that release into systemic circulation from lymphatic reservoirs influences overall oral bioavailability and first-pass drug metabolism of lymphatropic kinase inhibitors (Supplementary Fig. 12a–c). As metabolic decomposition of LP5-37 generates two equivalents of the PD315209 metabolite due to its chemical symmetry, the reciprocal levels of both LP5-37 and PD315209 observed among lymph node and blood (Supplementary Fig. 12d) further supports the lymphatic system as a controlled form of lymphatropic drug release into the systemic circulation. We propose this mode of absorptive drug release would reduce dose-limiting toxicities, providing for a diverse range of single-agent, combination and multi-targeted lymphatropic therapies capable of sustaining efficacious dosing ratios, to overcome compensatory signaling and resistance mechanisms in human diseases including cancer and autoimmune disorders.

## Discussion

Lymphatropic small molecule multi-targeted kinase inhibitors possess many distinctive attributes. The simultaneous ability to suppress regulatory pathways of cell survival, proliferation, and motility represents an opportunity to restrict uncontrolled tumor growth, cancer cell plasticity, and tumor heterogeneity; major therapeutic challenges due to signaling cross-talk and development of drug tolerance and resistance^47^. More specifically, PI3K and MAPK are amongst the most altered oncogenic signaling pathways in solid malignancies, and clinical efficiency using single-pathway inhibitors has been poor due to inadequate target suppression, activation of compensatory signaling, and convergent downstream targets^48^. LP-182 achieved selective and potent inhibition of PI3K and MAPK signaling pathways both in vitro and in vivo. Oral administration resulted in a prolonged plasma half-life, attributed to lymphatic absorption through active lipoprotein transport pathways in intestinal enterocytes. Release into systemic circulation from lymphatic reserves regulated LP-182 metabolic degradation and plasma concentration of potent metabolites LP-527 and PD0316684, reinforcing down-modulation of targeted signaling nodes. Notably, the metabolite analog PD0325901 has demonstrated significant pharmacologic activity against MAPK signaling at plasma concentrations of >50 ng mL^−1^, levels that were maintained for PD0316684 over 24 h upon LP-182 oral administration^49^. Likewise, sustained 20 ng mL^−1^ plasma levels of LP-527 were also observed following LP-182 oral dosing, concentrations which have been shown to down modulate PI3K signaling by the archetype GSK2126458 in breast cancer xenograft models^28^. By avoiding potential off-target effects and toxicity profiles associated with initial maximum serum concentrations (C_max_), these consistent and stable plasma concentrations within the therapeutic window are anticipated to extend the overall efficacy and therapeutic benefits of this class of lymphatically-directed inhibitors. Similar physiochemical and pharmacokinetic characteristics of additional multi-functional kinase inhibitors LP5-37, LP-616, and LP-65 to those of LP-182 signifies a generalized route for synthesis of lymphatropic small molecule kinase inhibitors with considerable therapeutic potential.

The central role of JAK/STAT signaling in MF has been established, but the use of JAK inhibitors has shown limited ability to produce durable remissions in most patient populations^50^. While genetic and functional studies have not fully defined the mechanisms for transformation and maintenance of the proliferative state in MF, targeting multiple signaling pathways will be required to alter the disease course and improve therapeutic outcomes^25,51–53^. Supportive of this assertion, compensatory MAPK activation through ligand-induced PDGFRα signaling was recently demonstrated to bypass JAK inhibition in animal models of MF. Combination of JAK and MEK inhibitors resulted in suppression of ERK activity in both *JAK2*^*V617F*^ and *MPL*^*W515L*^ mouse models, reducing splenomegaly and fibrosis, and improving overall efficacy as compared to JAK inhibition alone^27^. Synergism between JAK and PI3K inhibition has also been observed in cell and animal models of MPN, where combination treatments led to ~60% spleen weight reduction and extended overall survival compared to either inhibitor administered in isolation^54^. Interestingly, lone treatment with either PI3K or MAPK inhibitors did not result in a significant decrease in splenomegaly or most other MF phenotypes in the thrombopoietin receptor MPN animal models tested^27,54^. LP-182 treatment successfully down-regulated phosphorylation levels of both AKT and ERK in hematopoietic cells from *MPL*^*W515L*^ MF mice, preventing progression of splenomegaly, extramedullary hematopoiesis, and marrow fibrosis, to improve overall survival with no observed pharmacotoxicity. Thus, targeting of both PI3K and MAPK pathways with LP-182 alone or in combination with a JAK inhibitor provides a unique opportunity to potentially improve the clinical outcome of MF patients.

Myelofibrosis is characterized by irregular mobilization and trafficking of HSPCs through lymphoid tissue, establishing extramedullary sites of hematopoiesis within a permissive microenvironment^55^. Lymphatic tissues also serve as a conduit for cancer cells to escape primary tumors and metastasize to distant sites, or to remain sequestered within the favorable lymphatic milieu^56^. Furthermore, greater than 90% of the lymphocyte pool resides within the lymphatic system, approximately 50% of which are localized in the intestinal lymph and lymphoid tissues^57^. The ability to modulate targets using lymphatically-directed small molecules creates the prospect of forming an inhospitable environment to disrupt aberrant signal transduction, cellular trafficking, and immune cell networks, thereby reducing viability within the ‘protective’ lymphoid niche^58,59^. Thus, lymphatic targeting of lymphoid or cancer cells represent promising clinical applications for development of lymphatropic therapy to address challenges in auto-immunity and metastasis. We have developed a class of targeted small molecule kinase inhibitors that gain entrance into intestinal lymphatics intrinsically via lipid processing pathways. Further study will be required to more fully elucidate transport mechanisms along with the physiochemical properties required for orally bioavailable, lymphatically-absorbed compounds within a predictable chemical design framework. However, the presented synthetic medicinal chemistry strategy provides a flexible foundation along with motivation to stimulate further design of lymphatropic compounds for targeting single or multiple signaling pathways. While the inhibitor stoichiometry of LP-182 for PI3K and MAPK pathways is one-to-one, it is envisioned that mono-targeted lymphatropic agents could be formulated and dosed together in different proportions to provide tailored regimens based on individual compound pharmacokinetics. The archetypal design of LP-182 establishes a benchmark for development of lymphatropic compounds including drug repurposing^60^ in which existing drugs could be adapted for lymphatic delivery to provide innovative therapeutic opportunities.

## Methods

### Chemical synthesis and characterization

To develop single-molecule multi-functional kinase inhibitors, we required demonstration of simultaneous inhibition potency towards PI3K, mTOR, and MEK targets. For this purpose, we identified an analog, PD0316684, of the MEK inhibitor PD0325901^29^, and developed a novel dual PI3K/mTOR inhibitor analog of GSK2126458^28^, LP-527 (**7**), which allowed its chemical conjugation to PD0316684 via a multi-ethylene glycol linker (LP-622; 9b) to give LP-182. Additional investigative compounds described in this study were synthesized using similar methodology to that described in the synthesis of LP-182 (Supplementary Fig. 1a). In brief, MEK inhibitor compounds LP5-37 and LP5-38 were synthesized by conjugation of an analog of 8a with the appropriate ethylene glycol linker derivative using the method of Step h. The mTOR/MEK inhibitor LP-616 was synthesized from the carboxylic acid derivative of commercially available AZD2014^46^ (AZD2014CA) and an analog of 9b (wherein the pendant carboxylic acid is replaced by an amino group) using *N*-(3-dimethylaminopropyl)-*N*’-ethylcarbodiimide hydrochloride (EDC.HCl) and 1-hydroxybenzotriazole (HOBT) as coupling reagents. The mTOR/MEK inhibitor LP-65 was synthesized similarly through esterification of commercially available AZD8055^45^ with 9b (LP-622) using dicyclohexylcarbodiimide (DCC) and 4-(dimethylamino)pyridine (DMAP) under standard reaction conditions. Multi-ethylene glycol linker intermediates were obtained from Broadpharm (San Diego, CA). All other chemical reagents and anhydrous solvents were obtained from Aldrich Chemical Co. (Milwaukee, WI), and used without additional purification.

^1^H and ^13^C NMR spectra were recorded on Varian instruments at 400 and 500 MHz respectively, in DMSO-*d*_6_, CDCl_3_, or CD_3_OD as solvent and tetramethylsilane (TMS) as internal standard. Chemical shifts (δ) and coupling constants (*J*) are reported in parts per million (ppm) and Hertz (Hz), respectively. High resolution mass spectral analyses were performed at the Department of Chemistry, University of Michigan, using either a VG-70-250-S mass spectrometer for electron impact (EI) and chemical ionization (DCI) modes, a Waters Autospec Ultima instrument with an electrospray interface for electrospray ionization (ESI) mode, or a Waters Tofspec-2E run in reflectron mode. High performance liquid chromatography (HPLC) was performed using a Waters Breeze HPLC System (Waters Corporation, Milford, MA) equipped with a Waters 2487 Dual Wavelength UV Absorbance Detector. Analysis was conducted at ambient temperature on a Waters XSELECT CSH C-18 column (4.6 × 250 mm, 5 µm particle) using 0.1% TFA in H_2_O (A) and 0.1% TFA in CH_3_CN (B) solvent mixtures at a flow rate of 1 mL/min, and UV absorbance monitored at 254 and 280 nm. Separations were run over a 25 min linear gradient elution of either 30% B (initial) to 90% B (Method A) or 50% B (initial) to 90% B (Method B). All biologically tested compounds were demonstrated to have >96% chemical purity by reversed-phase gradient HPLC analysis.

*LP-182*; *N*-(2-(2-(2-(3-(4-(7-(6-chloro-5-((4-fluorophenyl)sulfonamido)pyridin-3-yl)quinoxalin-2-yl)piperazin-1-yl)-3-oxopropoxy)ethoxy)ethoxy)ethoxy)-3,4-difluoro-2-((2-fluoro-4-iodophenyl)amino)benzamide, ^1^H NMR (CD_3_OD): δ 8.67 (s, 1H), 8.43 (d, *J* = 2.0 Hz, 1H), 8.19 (d, *J* = 2.0 Hz, 1H), 7.90 - 7.86 (m, 3H), 7.80 (d, *J* = 2.0 Hz, 1H), 7.62 (dd, *J* = 8.5, 2.1 Hz, 1H), 7.38 (m, 1H), 7.35 (dd, *J* = 10.5, 2.0 Hz, 1H), 7.26 – 7.23 (m, 3H), 6.92 (br s, 1H), 6.51 (m, 1H), 4.01 (br s, 2H), 3.88 (br s, 2H), 3.79 (br s, 4H), 3.74 (br s, 4H), 3.68 (br s, 2H), 3.62 (m, 8H), 2.73 (m, 2H). ^13^C NMR (126 MHz, CD_3_OD): δ 167.7, 165.6, 155.3, 153.9, 153.4, 145.6, 144.4, 144.3, 144.2, 143.1, 139.5, 138.9, 138.4, 138.0, 137.9, 137.4, 137.1, 134.5, 133.7, 132.6, 131.2, 131.1, 130.1, 125.7, 125.5, 125.2, 125.1, 124.7, 120.8, 117.4, 117.2, 71.4, 71.2, 70.1, 68.6, 47.0, 46.6, 46.4, 45.7, 45.3, 42.6. HRMS (ESI^+^): *m/z* calculated for C_45_H_43_ClF_4_IN_8_O_8_S (M + H^+^), 1093.1588. Found: 1093.1585. HPLC (Method B): *t*_R_ = 14.5 min.

*LP-527 (7)*; *N*-(2-chloro-5-(3-(piperazin-1-yl)quinoxalin-6-yl)pyridin-3-yl)-4-fluorobenzenesulfonamide, ^1^H NMR (DMSO-*d*_6_): δ 8.85 (s 1H), 8.67 (br s, 1H), 7.94 - 7.90 (m, 2H), 7.80 - 7.77 (m, 3H), 7.62 - 7.61 (m, 1H), 7.56 (dd, *J* = 6.8, 1.6 Hz, 1H), 7.23 (t, *J* = 7.2 Hz, 2H), 3.99 (t, *J* = 4.0 Hz, 4H), 3.31 (br s, overlaps with HOD, 2H), 3.25 (t, *J* = 4.2 Hz, 4H). ^13^C NMR (126 MHz, DMSO-*d*_6_): δ 164.2, 162.2, 152.2, 143.8, 141.3, 139.4, 137.5, 136.4, 134.3, 129.5, 129.2, 124.0, 123.6, 115.7, 115.6, 43.2, 42.1. HRMS (ESI^+^): *m/z* calculated for C_23_H_21_ClFN_6_O_2_S (M + H^+^), 499.1114. Found: 499.1111. HPLC (Method A): *t*_R_ = 7.0 min.

*LP-622 (9b)*; 1-(3,4-difluoro-2-((2-fluoro-4-iodophenyl)amino)phenyl)-1-oxo-3,6,9,12-tetraoxa-2-azapentadecan-15-oic acid, ^1^H NMR (CDCl_3_): δ 10.25 (br s, 2H), 8.57 (br s, 1H), 7.41 - 7.37 (m, 2H), 7.30 (d, *J* = 6.8 Hz, 1H), 6.83 (m, 1H), 6.57 (m, 1H), 4.15 (br t, 2H), 3.77 - 3.71 (m, 4H), 3.67 - 3.64 (m, 4H), 3.60 (br m, 4H), 2.58 (t, *J* = 4.8 Hz, 2H). ^13^C NMR (126 MHz, CDCl_3_): δ 174.9, 154.3, 152.3, 144.3, 142.3, 132.9, 130.8, 130.7, 124.3, 124.2, 123.8, 119.9, 109.7, 109.6, 76.7, 75.1, 70.4, 70.2, 69.9, 69.6, 66.3, 53.4, 34.8. HRMS (ESI^+^): *m/z* calculated for C_22_H_25_F_3_IN_2_O_7_ (M + H^+^), 613.0653. Found: 613.0651. HPLC (Method A): *t*_R_ = 16.4 min.

### In vitro profiling of kinase inhibition

Quantitation of kinase inhibition was carried out by Life Technologies (Madison, WI) using the SelectScreen profiling platforms Z’-LYTE^TM^ and Adapta^TM^. Single-point broad panel kinome screening was performed at 2.5 µM LP-182 against all kinases within the Z’-LYTE^TM^ and Adapta^TM^ assays (321 kinase isoforms, holoenzymes, or mutant variants). The average percent kinase inhibition from replicate data was ranked low to high, converted to a compatible identifier format, then input into Coral Human Kinome Visualization software for presentation analysis^61^. Coral software does not recognize individual kinase holoenzymes or mutant variants, therefore the analysis represents the highest percent inhibition observed for a given kinase species in the primary screen. Coral software also does not recognize PI kinases, therefore this analysis was performed manually using the same scaling for branch color, node color, and node size. Scaling of evolutionary distance for PI kinases is only relative and is based on literature analysis^62,63^. Average percent kinome inhibition values from replicate data are supplied in Source Data file. Target kinase isoforms (PI3K, mTOR, RAF, MEK) were further assayed in at least replicate (*n* ≥ 2) against a 10-point curve of inhibitor concentrations for LP-182, LP-527, PD0316684, GSK2126458, and PD0325901 (Cayman Chemical). Corrected data was normalized to vehicle treated control values, and dose response percent kinase activity was analyzed by non-linear regression for determination of IC_50_ values.

### Molecular modelling and docking studies

Docking models of LP-182 were obtained using software from Schrödinger Inc. X-ray crystal structures of PI3Kγ (PDB code 3L08), mTOR (PDB code 4JSX), BRAF (PDB code 5HI2) and MEK1 (PDB code 3ORN)^28,31–33^ were selected based on native ligand similarity to LP-182 and prepared using the Protein Preparation Wizard in Maestro (Protein Preparation Wizard, Schrödinger, LLC, New York, NY). Protein structures were used to generate receptor grids centered on the native ligand docking sites with a maximum ligand length of 36 Å using OPLS3 due to the larger size of the LP-182 ligand. LP-182, LP-527, and PD0316684 inhibitor ligands were built and prepared for docking in Maestro using LigPrep 3.8 (LigPrep, Schrödinger, LLC, New York, NY). The docking procedures were performed using Glide 7.1 in standard precision mode with default parameters and no constraints. Generated poses for each protein-ligand structure were filtered by docking score of ≤ −2.0 and curated manually, with top poses of LP-182 selected based on their chemical and positional alignment with both native ligand and either LP-527 (PI3Kγ and mTOR) or PD0316684 (BRAF and MEK1), and overall docking score (≤−5.3 for all poses presented). Two-dimensional ligand interaction diagrams were generated from the selected poses using the Schrödinger software tools to highlight protein-ligand interactions. All diagrams were processed to present the best 2-D orientations, rotated to align ligand structures among diagrams, then cleaned up to normalize orientation and arrangement of groups using the ligand interaction diagram toolbar functions.

### Flow cytometry analysis

The human megakaryoblastic cell line, SET-2 (ATCC), was maintained in RPMI 1640 supplemented with 20% FBS and incubated for 16 h at 10^6^ cells mL^−1^ with LP-182 or vehicle (DMSO; dimethyl sulfoxide), then washed in ice-cold PBS, and collected for flow cytometry analysis. Single-cell suspensions of mouse spleen were prepared from *MPL*^*W515L*^ MF mice (below) in RPMI 1640 supplemented with 5% FCS. Red blood cells were lysed by ammonium chloride-mediated red cell lysis (RCL) buffer and passed through a nylon mesh, and then cells were counted. For intracellular phospho-protein staining, SET-2 or spleen cells were fixed and then permeabilized using BD Phospho-flow fixation buffer and Perm-Buffer III (BD biosciences) according to the manufacturer’s protocol. Intracellular phospho-protein staining was performed using fluorochrome-conjugated antibodies, pAKT (pS473)-Alexa Fluor 647 (MB89-61) or pERK1/2 (pT202/pY204)-Alexa Fluor 647 (20 A). All phospho-flow antibodies were purchased from BD Biosciences. Antibodies for surface staining were purchased from Biolegend; CD3e-BV510 (145-2C11), CD11b-APC-Cy7 (M1/70), CD19-BV605 (6D5), Ly-6G-PE-Cy7 (1A8), TER-119-BV650 (TER-119), CD71-PerCP-Cy5.5 (RI7217), CD117-AlexaFluor-700 (2B8), Sca-1-PE/dazzle 594 (E13-161.7), CD41-PE (MWReg30), CD11c-APC (N418), F4/80-BV605 (BM8), CD150-BV650 (TC15-12F12.2), CD41-Percp-Cy5.5 (MWReg30). Flow cytometry was performed on a FACS Fortessa (Becton, Dickinson and Co.) using FACSDiVa software, and data analyzed using FlowJo software (Tree Star). Gating strategy is outlined in Supplementary Fig. 9. In vitro dose response data for AKT and ERK1/2 phosphorylation was normalized to vehicle treated control cells. Data was generated from *n* = 3 independent experiments or *n* ≥ 3 individual animals.

### Proliferation and Caspase activation assays

For proliferation assays, SET-2 cells were cultured for 72 h at 10^6^ cells mL^−1^ with various concentrations of LP-182, LP-527, PD0316684 or vehicle (DMSO). Viable cells were counted by CellTiter-Fluor Assay (Promega) according to manufacturer’s protocol, and all fluorescence values were corrected by a no-cell control. For Caspase activation assays, SET-2 cells were cultured for 48 h at 10^6^ cells mL^−1^ with various concentration of LP-182 or vehicle (DMSO). Caspase activity was measured by Caspase-Glo 3/7 assay (Promega) according to the manufacturer’s protocol, and all luminescence values were corrected by a no-cell control. Dose response proliferation and Caspase activation data was normalized to vehicle treated control cells and analyzed by non-linear regression for determination of IC_50_ values. Data was generated from *n* = 3 independent experiments performed in replicate.

### Animal studies

Adult (6–8 week old) female CD-1 and BALB/c mice, and adult male Sprague-Dawley rats (~300–400 g), were purchased from Charles River Laboratories (Wilmington, MA). All animal procedures were approved by the University of Michigan Institutional Animal Care and Use Committee and were performed in compliance with all relevant ethical regulations therein. All rodents were maintained in a specific pathogen-free barrier unit at the University of Michigan accredited by the Association for Assessment and Accreditation of Laboratory Animal Care. All orally administered compounds were dissolved in Maisine^®^ CC (Gattefossé; Saint-Priest, France) and administered by gavage with a water chase unless otherwise specified.

### Pharmacokinetic analysis

Quantitative determination of LP-182, LP-616, and LP-65 blood concentration over time, along with identified metabolites, was performed by the University of Michigan Pharmacokinetics Core. Two groups of female CD-1 mice (*n* = 9 per group per compound) were divided for intravenous dosing at 15 mg kg^−1^ (in PBS containing 20% DMSO and 50% PEG-400) and oral dosing (in Maisine CC with water chase) at 400 mg kg^−1^ (LP-182) or 200 mg kg^−1^ (LP-616 & LP-65), respectively. Blood samples were collected using heparinized calibrated pipettes across 10 time points (0.083, 0.167, 0.25, 0.5, 1, 2, 4, 7, 16, and 24 h) for intravenous and 9 time points (0.167, 0.25, 0.5, 1, 2, 4, 7, 16, and 24 h) for oral administration over a 24 h period (*n* ≥ 3 animals sampled per time point per compound dosing). Samples were centrifuged at 15,000 rpm for 10 min, then blood plasma was collected from the upper layer and frozen at −80 °C until analysis. LP-182, LP-616, LP-65, and their metabolites, LP-527, PD0316684, LP-622, PD315209, AZD2014CA, LP6-26, and AZD8055 were separated and analyzed by reverse-phase chromatography using an XBridge C18 column (50 mm × 2.1 mm i.d., 3.5 µm; Waters Corp.) flowing binary mobile phase gradients of 0.1% formic acid in water and 0.1% formic acid in acetonitrile on an Axion LC system coupled to an AB Sciex 4000 QTRAP mass spectrometer. Sample processing, chromatographic separation, MS, and MS/MS parameters used for analysis were determined by the core. Standard curves were generated from purified compounds and internal standard (CE302) spiked in equivalent biological matrix, having a combined average accuracy and precision measurement across three concentrations of each parent compound of 102.8% ± 5.8% RSD, respectively. Standard curves for peak area ratio of compound to internal standard (A_std_/A_Istd_) vs. compound concentration were used to determine sample analyte concentrations through linear regression with a weighting average of 1/X^2^. All standard curves displayed a correlation coefficient of r^2^ ≥ 0.991 and a linear dynamic range in between 1 ng mL^−1^ and 10,000 ng mL^−1^ depending on the specific compound or metabolite analyzed. All pharmacokinetic parameters were estimated using non-compartmental analysis with Phoenix/WINONLIN, and provided by the core along with calculated sample analyte concentrations. Data was generated from *n* ≥ 3 individual animals sampled per time point per compound for each method of dosing.

### Microsomal stability

Quantitative determination of LP-182 stability in murine liver microsomes was performed by the University of Michigan Pharmacokinetics Core. Briefly, LP-182 or control compound Verapamil (in 1–10% DMSO) were mixed to a final concentration of ~1 µM (~0.1–1% DMSO) with murine liver microsomes (~0.5 mg mL^−1^ in 100 mM phosphate buffer, 3 mM MgCl_2_), then warmed at 37 °C for 3 min. Reactions were initiated by addition of nicotinamide adenine dinucleotide phosphate (NADPH; in 100 mM phosphate buffer, 3 mM MgCl_2_) to a final concentration of 1 mM, and then reactions were sampled over 60 min. Aliquots were removed at the designated time points, quenched by addition of 4 volumes of cold acetonitrile, then centrifuged at 10,000 x *g* for 5 min to pellet protein precipitates. The supernatant of extracted samples was injected (5 µL) and separated by reverse-phase chromatography using an ACQUITY BEH C18 column (50 mm × 2.1 mm i.d., 1.7 µm; Waters Corp.) flowing binary mobile phase gradients of 0.1% formic acid in water and 0.1% formic acid in acetonitrile at 0.5 mL min^−1^, and analyzed by MS. All sample processing, chromatographic separation, MS, and MS/MS parameters used for analysis were determined by the core as outlined in the Methods section of the manuscript. The natural log peak area ratio of compound to internal standard (A_cmpd_/A_Istd_) was plotted against time to determine pharmacokinetic properties of microsomal stability. All analyses, data processing, and calculations were performed and provided by the core. Data was generated from a single independent experiment.

### Bioavailability studies

Seven groups of female CD-1 mice (*n* = 4 per dosing group) received a single oral dose of LP-182 at 0, 100, 200, 400, 600, 800 and 1000 mg kg^−1^ solubilized in Maisine CC. Incomplete solubilization of LP-182 was observed at >600 mg kg^−1^ so suspensions were administered for these concentrations. Urine and fecal samples were collected over 24 h at 24, 48 and 72 h intervals using metabolic cages, and quantitation of LP-182 in each sample was determined by the University of Michigan Pharmacokinetics Core. Briefly, water was added to fecal samples at an average ratio of 2.5 ± 1.6 (w/w) water:feces to generate fecal homogenates using a tissue homogenizer (Omni International TH), then approximately 200 mg of homogenate was diluted 5-fold with 20% (v/v) acetonitrile in water prior to analysis. All sample processing, chromatographic separation, MS, and MS/MS parameters used for analysis were determined by the core as outlined in the Methods section of the manuscript. Standard curves were generated from purified compounds and internal standard (CE302) spiked in equivalent biological matrices to determine sample analyte concentrations as above, with all curves displaying a correlation coefficient r^2^ ≥ 0.992 and a linear dynamic range in between 0.01 µg mL^−1^ and 100 µg mL^−1^ depending on the specific matrix, dosing, and time point analyzed (24, 48, or 72 h). The cumulative amount of compound recovered over 72 h in feces and urine was determined from sample analyte concentrations provided by the core, then converted to percent recovered by normalization to the amount of compound administered. Percent oral bioavailability was determined as the percent difference between the amount of compound administered to the amount of compound recovered over 72 h in feces. LP-182 dose (mg kg^−1^):LP-182 administered (mg) is 100:2.7; 200:5.4; 400:10.8; 600:16.2; 800:21.6; 1000:27.0. Data was generated from n = 4 individual animals per compound dose.

### Compound quantitation in blood and mesenteric lymph nodes

Female CD-1 (*n* ≥ 4 per compound per study grouping) were orally dosed with LP-182, LP-527, PD0316684, LP5-38, LP5-37, LP-616, or LP-65 at concentrations ranging from 100 mg kg^−1^ to 1000 mg kg^−1^ and sacrificed according to animal protocols. Solubilization of LP-527 (100 mg kg^−1^) and LP-182 (>600 mg kg^−1^) in Maisine CC was incomplete, so suspensions were administered for these compound dosages. Whole blood and mesenteric lymph nodes were collected either 30 min or 4 h following compound administration and stored at −20 °C until future processing and analysis by the University of Michigan Pharmacokinetics Core as above, or as outlined below, to determine compound concentrations in each tissue. For analyses performed by the core, sample processing, chromatographic separation, MS, and MS/MS parameters used were determined therein, and calculated sample analyte concentrations provided. Here, standard curves were generated from purified compounds and internal standard (CE302) spiked in equivalent biological matrices to determine sample analyte concentrations as above, with all curves displaying a correlation coefficient r^2^ ≥ 0.997 and a linear dynamic range in between 10 ng mL^−1^ and 20 µg mL^−1^ depending on the specific matrix, dosing, and compound analyzed. Data was generated from *n* ≥ 4 individual animals per compound per study group or dose. A single data set presented in Fig. 3b is also represented as a ratio in Fig. 6b (PD0316684, 100 mg kg^−1^, 4 h). One animal was excluded from blood and mesenteric lymph node analysis (LP-182, 400 mg kg^−1^, 4 h; Fig. 6e and associated figures) as an identified outlier based on testing by ROUT method (Q = 0.1%).

### Rat mesenteric lymph duct cannulation model

A rat mesenteric lymph duct cannulation model was used to collect and quantify LP-182 in lymphatic fluid, blood, and lymphatic proteo-lipid sub-fractions similar to previously described methods^37,40^. Male Sprague-Dawley rats (*n* = 7) were anesthetized by isoflurane (Piramel, PA) and placed on a heated pad maintained at 37 °C using a Kent Scientific (Torrington, CT) low flow vaporizer and SomnoSuite for pulse oximetry and thermal regulation, respectively. The carotid artery, mesenteric lymph duct and duodenum were cannulated, and normal saline was continuously infused into the duodenum at 1.7 mL h^−1^ using a constant rate infusion pump. After approx. 0.5 h, 50 mg kg^−1^ of LP-182 solubilized in ~1.7 mL of either Maisine CC or a mixture of 93% Maisine CC and 7% Labrasol (Gattefossé; Saint-Priest, France) was infused over 1 h into the duodenum, followed by normal saline (as above) for the duration of the experiment. Hourly fractions of lymphatic fluid were collected continually (rate of ~0.5–1.2 mL h^−1^) for approx. 6 h, while arterial blood samples (10–20 µL) were collected once per hour and stored at −20 °C for future analysis. Lymphatic fluid time course samples were either frozen or pooled for subsequent fractionation by ultracentrifugation to isolate proteo-lipid sub-fractions similar to previously described methods^39^. Separation of chylomicrons was achieved by layering lymphatic fluid under PBS (Gibco; ThermoFisher Scientific) in a polyallomer ultracentrifuge tube (Beckman, CA), and centrifugation in a L8-70M ultracentrifuge (Beckman, CA) at 202,048 x g using a SW40Ti rotor (Beckman, CA) for 95 min at 15 °C. Following centrifugation, the semi-solid chylomicron layer at the top of the centrifuge tube was recovered and stored at −20 °C for future analysis. After replacing the void volume with PBS, the bottom layer underwent further ultracentrifugation for an additional 16 h as above, at which time a viscous dispersion of VLDL was collected from the top of the tube, and an aqueous fraction containing LDL/HDL from the bottom, which were stored at −20 °C for future analysis. All lymphatic fluid, blood, and fractionated chylomicron, VLDL, and LDL/HDL samples were analyzed by mass spectrometry for quantitation of LP-182 as outlined below. Relative LP-182 in lymphatic fluid proteo-lipid sub-fractions was calculated as the amount of compound in a specified fraction as a percentage of the cumulative amount of compound measured in all fractions. Data was generated from *n* ≥ 3 individual animals sampled per time point per study condition. Relative LP-182 in blood was calculated as the amount of compound measured in a specified temporal fraction of blood as a percentage of the amount of compound measured in an equal volume of the same temporal fraction of lymphatic fluid, with the average calculated from all fractions. Data was generated from *n* = 2 individual animals sampled per time point per study condition due to limitations in blood volume collection.

### Liquid chromatography tandem mass spectrometry (LCMS/MS)

LP-182, LP-527, PD0316684, LP5-38, LP5-37, LP-616, LP-65, LP-622, PD315209, AZD2014CA, LP6-26, and AZD8055 were analyzed using an ACQUITY UPLC H-Class (Waters Corp.) coupled to a SCIEX 5500+ QTRAP mass spectrometer (AB Sciex). Quantitation was performed against standard curves generated from purified compounds and internal standard (Aripiprazole-*d*8; Cerilliant Corp.) spiked in equivalent biological matrice(s), having a combined average accuracy and precision measurement across multiple concentrations of each compound of 102.9% ± 10.1% RSD, respectively. Standards and compound extraction from biological fluids or tissues was performed using similar protocols, all resulting in recoveries ≥90%. Briefly, acetonitrile was added to a final concentration of 60–80% to bone marrow, whole blood, plasma, lymphatic fluid, lymphatic proteo-lipid sub-fractions, or mesenteric lymph node samples for compound extraction. Extraction was performed by shaking and/or bead rupture using a Precellys Evolution tissue homogenizer (Bertin Technologies SAS). Extracted samples were centrifuged at 10,000 x *g*, supernatant transferred to autosampler vials, and then separated by reverse-phase chromatography using an ACQUITY BEH C18 column (50 mm × 2.1 mm i.d., 1.7 µm; Waters Corp.) flowing binary mobile phase gradients of 0.1% formic acid in water and 0.1% formic acid in acetonitrile (Optima LC-MS Grade; Fisher Scientific) at 0.6 mL min^−1^, optimized to achieve baseline separation of all compounds. Optimization of MS and MS/MS parameters for MRM quantitation and maximum sensitivity of individual compounds was performed using SCIEX Analyst software (AB Sciex). Standard curves for peak area ratio of compound to internal standard (A_std_/A_Istd_) vs. compound concentration were used to determine sample analyte concentrations through linear regression with a weighting average of 1/X. All standard curves displayed a correlation coefficient r^2^ ≥ 0.994 and a linear dynamic range in between 0.075 ng mL^−1^ and 160 ng mL^−1^ depending on the specific matrix, dosing, and compound/metabolite analyzed. All LC-MS/MS acquisitions were performed with an independent standard curve for each group of analytes and for each individual run. All solvents utilized were of Optima LC-MS Grade (Fisher Scientific). Data was generated from *n* ≥ 2 individual animals per compound per study condition or dose.

### *MPL*^*W515L*^ MF mouse model

Generation of the *MPL*^*W515L*^ mutant MF mouse model was similar to previously described methods^27^. Briefly, female donor BALB/c mice were treated with 5-fluorouracil at 150 mg kg^−1^ intraperitoneal for 5 d before harvesting bone marrow. Hematopoietic stem and progenitor cells were enriched with CD117 magnetic beads (Miltenyi Biotec), transduced with retrovirus containing MSCV-hMPL W515L-IRES-GFP, then 50,000 transduced HSPCs along with 300,000 total non-stem cells were injected intravenously into lethally irradiated female BALB/c recipients. Development of MF was evaluated by spleen volumes using MRI beginning ~14 d following bone marrow transplantation (BMT) as outlined below, continuing weekly until end of the study. Mice were separated into equal groups based on spleen size ~14–21 d post-BMT, then treated with vehicle (Maisine CC) or LP-182 at 400 mg kg^−1^ daily by oral gavage for 14–28 d or until mice were euthanized using guidelines for end stage illness and humane endpoints. Untreated healthy control animals were used for experimental comparisons. Tissues were harvested and processed for flow cytometry (as described above), LCMS/MS, immuno-blotting, and histological analysis (as described in Supplementary Methods) at the defined study endpoint(s), approximately 1–2 h post-treatment with vehicle or LP-182. Briefly, blood samples were collected, transferred into K_2_-EDTA tubes (BD Microtainer), inverted gently to mix, then centrifuged at 2000 x *g* for 15 min. Blood plasma was collected from the upper layer and frozen at −80 °C until analysis by LCMS/MS (as described above). Dissection and bone marrow isolation from mouse femur and tibia was performed similar to previously described methods^64^. Briefly, the hind limbs from mice were dissected, muscle and connective tissue removed, and bones were frozen at −80 °C. Femur and tibia were thawed separately, placed knee-end down in 18 gauge needle-pierced 0.5 mL microcentrifuge tubes nested inside 1.5 or 2 mL microcentrifuge tubes containing 1.4 mm ceramic beads (Fisher Scientific), then bone marrow was isolated by centrifugation at 10,000 x *g* for 1 min and processed for analysis by LCMS/MS (above; femur) or immuno-blotting (tibia). Bone marrow tissue (tibia) was homogenized in 1.25X RIPA buffer (62.5 mM HEPES pH 7.4, 187.5 mM NaCl, 1.25X cOmplete protease inhibitor cocktail and 1.25X PhoSTOP phosphatase inhibitor cocktail (Roche), 1 mM AEBSF, 10 µM E-64) by bead rupture (as above), then cells lysed by addition of detergent (to final concentration of 1X RIPA, 1% NP-40, 0.5% sodium deoxycholate, 0.1% SDS) and incubation rotating at 4 °C for 30 min. Soluble protein extracts were isolated by centrifugation at 15,000 x *g* for 15 min at 4 °C, quantified using Micro BCA protein assay (Thermo Scientific), then mixed with reducing 6X SDS-PAGE loading dye, heated for 8 min at 95 °C, and stored at −20 °C until immuno-blot analysis. Whole cell lysates were resolved by 10% SDS-PAGE, transferred to PVDF (Immobilon-P; MilliporeSigma), then membranes were blocked with 5% BSA (Equitech-Bio) in TBS containing 0.1% Tween-20 (TBST) for 1 h at room temperature with gentle orbital shaking. Membranes were incubated with primary antibodies in 5% BSA/TBST overnight at 4 °C or 5% skim milk/TBST (Lab Scientific) for 1 h at room temperature, then washed with TBST, and incubated with HRP-conjugated secondary antibodies in 5% skim milk/TBST for 1.5 h at room temperature, all with gentle platform rocking. Membranes were washed with TBST, then visualized by chemiluminescence on a BioRad ChemiDoc MP Imaging System using SuperSignal West PicoPLUS or West Femto Chemiluminescent Substrate (Thermo Scientific). Membranes were stripped using Restore PLUS Western Blot Stripping Buffer (Thermo Scientific) as per manufacturer’s instructions and re-probed with additional primary antibodies as described above. Antibodies and blotting reagents used in this study include the following: mouse monoclonal anti-GAPDH (1D4, NB300-221; Novus Biologicals); rabbit polyclonal anti-phospho-p44/42 MAPK(Erk1/2)(Thr202/Tyr204) (9101; Cell Signaling Technology); anti-p44/42 MAPK(Erk1/2) (9102; Cell Signaling Technology); goat polyclonal anti-GFP (600-101-215; Rockland Immunochemicals); anti-mouse-IgG(H + L)-HRP conjugate (155-035-003; Jackson ImmunoResearch Inc.); anti-rabbit-IgG(H + L)-HRP conjugate (111-035-003; Jackson ImmunoResearch Inc.); donkey polyclonal anti-goat-IgG(H + L)-HRP (705-035-003; Jackson ImmunoResearch Inc.). All antibodies were used at dilutions recommended by their specified manufacturers or optimized empirically. Additional reagents were purchased from MilliporeSigma (St. Louis, MO) unless otherwise specified. Tissues for histology were formalin-fixed, paraffin-embedded, sectioned, and stained with Hematoxylin and Eosin (H&E) to assess cellularity and tissue morphology, with Reticulin Stain Kit (Connective Tissue Stain; Abcam) to assess fibrosis, or processed for immunohistochemistry staining with GFP to assess *MPL*^*W515L*^-positive cellularity. Digital images of tissue sections were obtained using an Olympus BX 43 microscope (Olympus Corp.), and adjusted using Adobe Photoshop for viewing. Quantitation of megakaryocytes was from the average of 5 high-power fields (40x) per individual animal, with prioritization of cell clusters and consistent regional staining (edge of spleen) for GFP-positive megakaryocyte quantitation. Reticulin fibrosis in bone marrow of the femur was scored based on the WHO grading system^65,66^. Extramedullary hematopoiesis (EMH) in the liver and spleen was based on comparative subjective assessment. Individual data sets and scoring details are provided in Supplementary Table 1. Data was generated from *n* ≥ 3 individual animals per study condition. Two vehicle treated animals were excluded entirely from analysis due to procedural complications requiring euthanasia (Study 1) and severe loss of body weight beginning 7 d after experiment initiation and observed liver discoloration upon organ harvest (Study 2). Two animals were excluded from immune cell number analysis (Study 1) due to inability to collect sample (vehicle) and poor data quality (LP-182), respectively. One animal was excluded from GFP-positive megakaryocyte quantitation and immuno-blotting (Study 1) due to poor/absent positive immunohistochemistry staining and low MK cell numbers (LP-182). The MSCV-hMPL W515L-IRES-GFP expression vector was a generous gift from Ann Mullally (Brigham and Women’s Hospital, USA).

### Magnetic resonance imaging

MRI was performed on a Bruker BioSpin console running ParaVision version 7.0 software on a 30 cm diameter horizontal-bore Magnex 7 T magnet (300 MHz ^1^H frequency) equipped with a ^1^H 72 mm transmit/receive coil for spleen imaging, and a closed-cycle cryogenically cooled 4-element surface receive coil and preamplifier for imaging the tibia. Spleens and tibias were imaged in separate scan sessions. A two-dimensional multi-slice FLASH sequence was used to obtain abdominal scans for spleen volume imaging. Acquisition parameters were as follows: repetition time/echo time (TR/TE) = 600/8 ms, 4 averages, flip angle = 30°, slice thickness = 0.4 mm, field of view = 40 × 30 mm^2^, 40 slices, matrix = 256 × 128, total acquisition time = 154 s. Respiratory gating was achieved using triggering through a small animal monitoring system with pneumatic pillow device (SA Instruments, NY) to reduce motion artifact. Spleen volumes were obtained from the multi-slice MR images. Organ boundaries visualized in each slice were manually segmented using publicly available image visualization software (www.slicer.org). The quantity of pixels within segmented spleen, summed over all slices and multiplied by known pixel volume (0.0146 mm^3^) yielded spleen volume. Spleen volumes were normalized to individual animal body weights or spleen weights taken no more than 4 d or 1 d from each scan date, respectively. Apparent diffusion coefficient (ADC) within the tibia marrow space was measured by MRI using the following acquisition parameters: diffusion-weighted scan with TR/TE = 2000/30 ms, 2 averages, field of view = 23.0 × 9.6 mm^2^, matrix = 128 × 64, slice thickness = 0.150 mm, 40 slices, nominal *b* values of 0 and 1000 s/mm^2^, total acquisition time = 17 min. For bone marrow analysis of vehicle and treated mice at defined time points, spatial registration of all tibia image data sets to baseline data was performed. Briefly, volumes-of-interest (VOIs) were manually segmented within the tibia bone marrow space at 0 d treatment for each individual animal and applied as a mask in a full-affine transformation using publicly available *Elastix* software (version 5.0.1) (https://elastix.lumc.nl/) to volumetrically align serial tibia image sets to a common geometric space^67^. To standardize measurements across animals, the axial slice near the proximal end of the tibia containing the largest cross-sectional marrow space (i.e. slice with largest number of pixels within the VOI) provided the landmark aligned across all animals such that linear location throughout the entire length of the tibia was consistently measured relative to this proximal landmark. The distal tibia bone marrow space used to generate multi-slice average ADC measurements was defined as 4.5 mm from the proximal landmark to the end of the VOI. The ADC values for each slice within this distally-defined region were averaged for each individual animal then used in subsequent comparisons among the treatment groups. Data was generated from n ≥ 4 individual animals per study per condition.

### Pathological and histological toxicity screening

Female CD-1 mice were dosed orally with vehicle (Maisine CC; *n* = 5) or LP-182 at 400 mg kg^−1^ (*n* = 4) daily for 10 d, followed by an 8 d rest period, then underwent necropsy for hematological, histological, and pathological evaluation by the University of Michigan Unit for Laboratory Animal Medicine In Vivo Animal Core. Untreated animals (*n* = 3) were also monitored during the study for non-compound related adverse findings (ie. gavage stress impacting weight gain, vehicle-related toxicity), however no findings were observed, thus main comparisons were made between vehicle and compound treated mice. Mice were assessed unblinded by a board-certified veterinary pathologist for previously reported toxicities caused by the dual PI3K/mTOR inhibitor GSK2126458 such as anemia, neutropenia, stomatitis, hepatotoxicity, and pneumonitis, as well as for any other adverse findings, using standard procedures for hematology, gross evaluation of body and organ weights, and histopathology of major organs^68–70^. Briefly, mice were euthanized by CO_2_ inhalation, then blood was collected by cardiac puncture and transferred into EDTA anticoagulant-containing tubes, followed by gross necropsy, then tissue harvest and fixation in 10% neutral buffered formalin at a 10:1 (v/v) ratio of fixative to tissue. Differential blood counts were generated from whole blood using a Hemavet 950 automated hematology analyzer (Drew Scientific), and tissues were processed for paraffin embedding using routine histological methods, then sectioned at 4 µm by rotary microtome and mounted on glass slides for hematoxylin and eosin (H&E) staining and evaluation by light microscopy at magnifications ranging from 10–40x. Data was generated from *n* ≥ 3 individual animals per grouping. Two animals in the vehicle treated group were excluded from blood count analysis due to insufficient sample collection and sample clotting, respectively.

### Statistical analysis

All statistical tests were performed using GraphPad Prism (version 8.0.0; GraphPad Software Inc., La Jolla, CA, USA). Individual figure legends describe statistical testing and values.

### Reporting summary

Further information on research design is available in the Nature Research Reporting Summary linked to this article.

## Supplementary information

Supplementary Information

Reporting Summary

## Data Availability

Data that support the findings of this study are available within the article and its Supplementary Information. Source data are available as a Source Data file. Source data are provided with this paper.

## Source data

Source Data
