# Supplementary material for: A lymphatic-absorbed multi-targeted kinase inhibitor for myelofibrosis therapy
Source: Nat Commun. 2022 Aug 17;13:4730. doi: 10.1038/s41467-022-32486-8 (PMC9386018; doi:10.1038/s41467-022-32486-8)
Supplement: Supplementary file 1 — Supplementary Information [file 41467_2022_32486_MOESM1_ESM.pdf]

Supplementary Information for

A lymphatic-absorbed multi-targeted kinase inhibitor for myelofibrosis therapy

Brian D. Ross[1,2*], Youngsoon Jang[1], Amanda Welton[1], Christopher A. Bonham[1], Dilrukshika S.W. Palagama[1], Kevin Heist[1], Jagadish Boppisetti[1], Kasun P. Imaduwage[1], Tanner Robison[1,3], Leah R. King[1], Edward Z. Zhang[1], Cyrus Amirfazli[1], Kathryn E. Luker[1], Winston Y. Lee[4], Gary D. Luker[1,2,5], Thomas L. Chenevert[1], Marcian Van Dort[1]

*Correspondence to: bdross@umich.edu

This PDF file includes:

Supplementary Figs. 1-12
Supplementary Table 1
Supplementary References

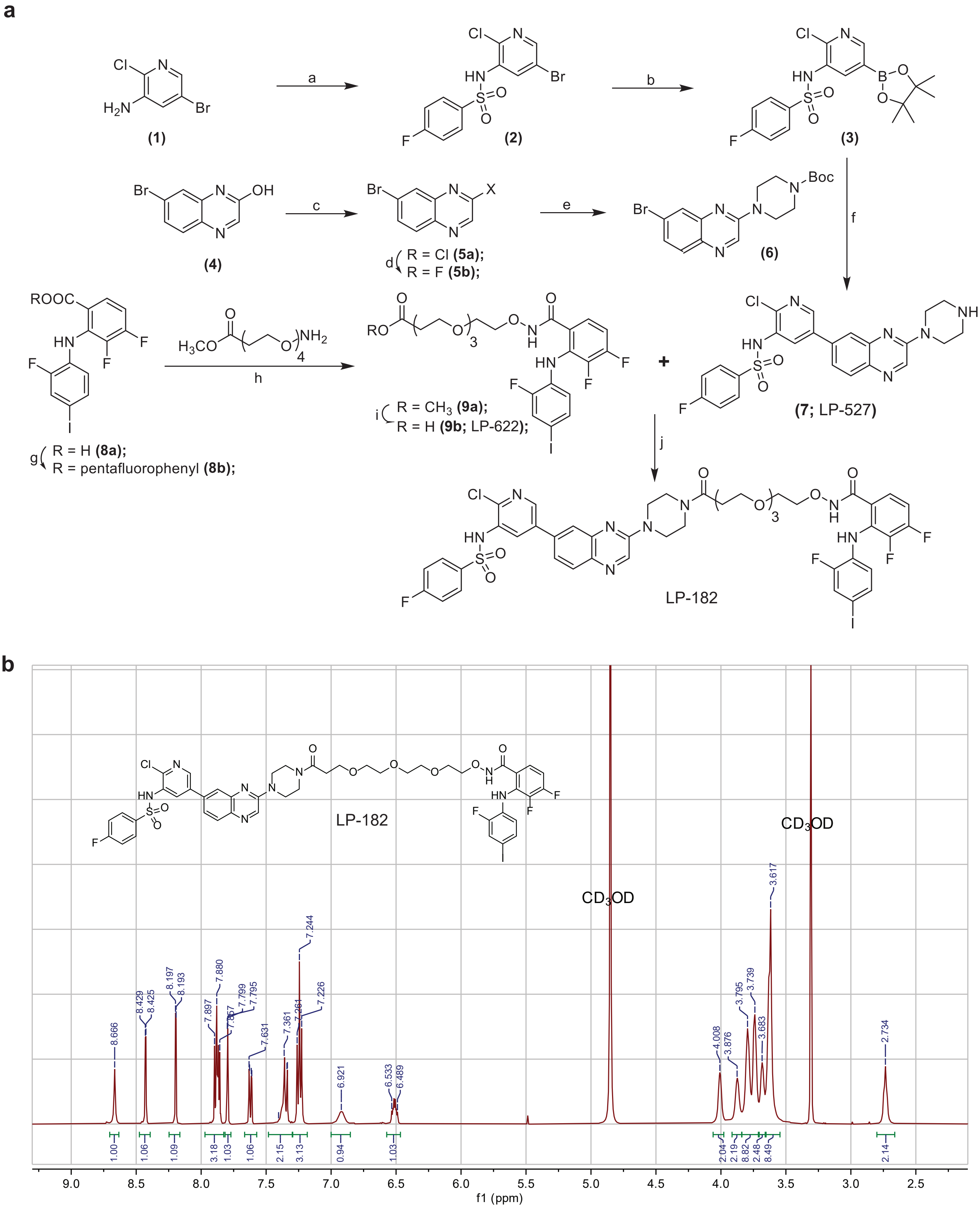

Supplementary Figure 1. **Synthesis and characterization of LP-182. a** Synthetic scheme and conditions for LP-182. (a) 1. 4-fluorophenylsulfonyl chloride, pyridine, 100 °C; 2. K_2CO_3, CH_3OH, H_2O, RT; (b) bis(pinacolato)diborane, KOAc, $PdCl_2$dppf, dioxane, 120 °C; (c) $POCl_3$, toluene, RT; (d) TBAF, DMSO, RT; (e) *N*-Boc piperazine, THF, reflux; (f) 1. SilicaCat DPP-Pd, 2M $CsCO_3$, 100 °C; 2. HCl, dioxane, THF, 40 °C; 3. basic workup/extraction; (g) pentafluorophenol trifluoroacetate, pyridine, DMF, RT; (h) Et3N, THF, RT; (i) 1N LiOH, MeOH, RT; (j) pivaloyl chloride, Hunigs base, DCM:THF, RT. **b** ^{1}H NMR spectra and associated NMR peak assignments for LP-182; *N*-(2-(2-(2-(3-(4-(7-(6-chloro-5-((4-fluorophenyl)sulfonamido)pyridin-3-yl)quinoxalin-2-yl)piperazin-1-yl)-3-oxopropoxy)ethoxy)ethoxy)ethoxy)-3,4-difluoro-2-((2-fluoro-4-iodophenyl)amino)benzamide.

a

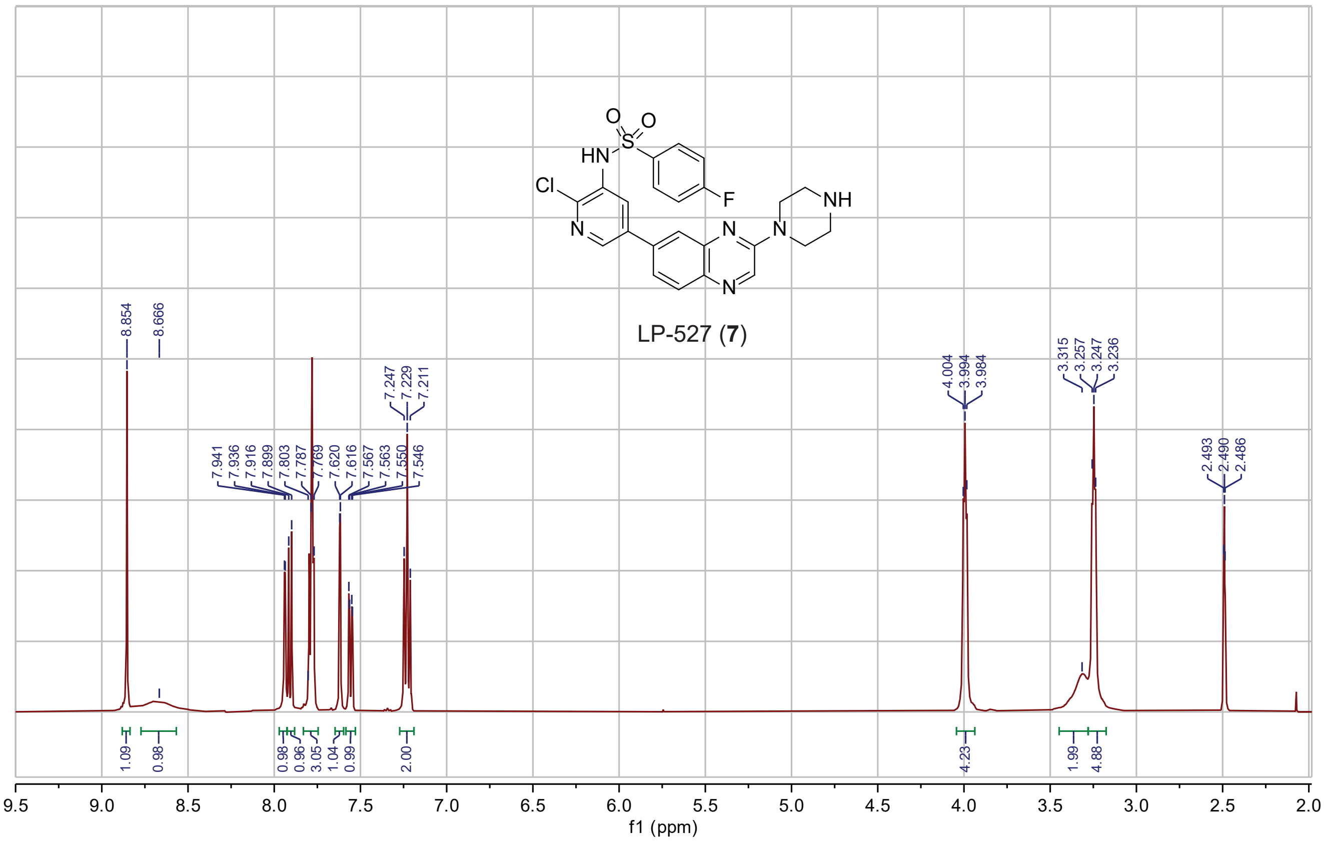

b

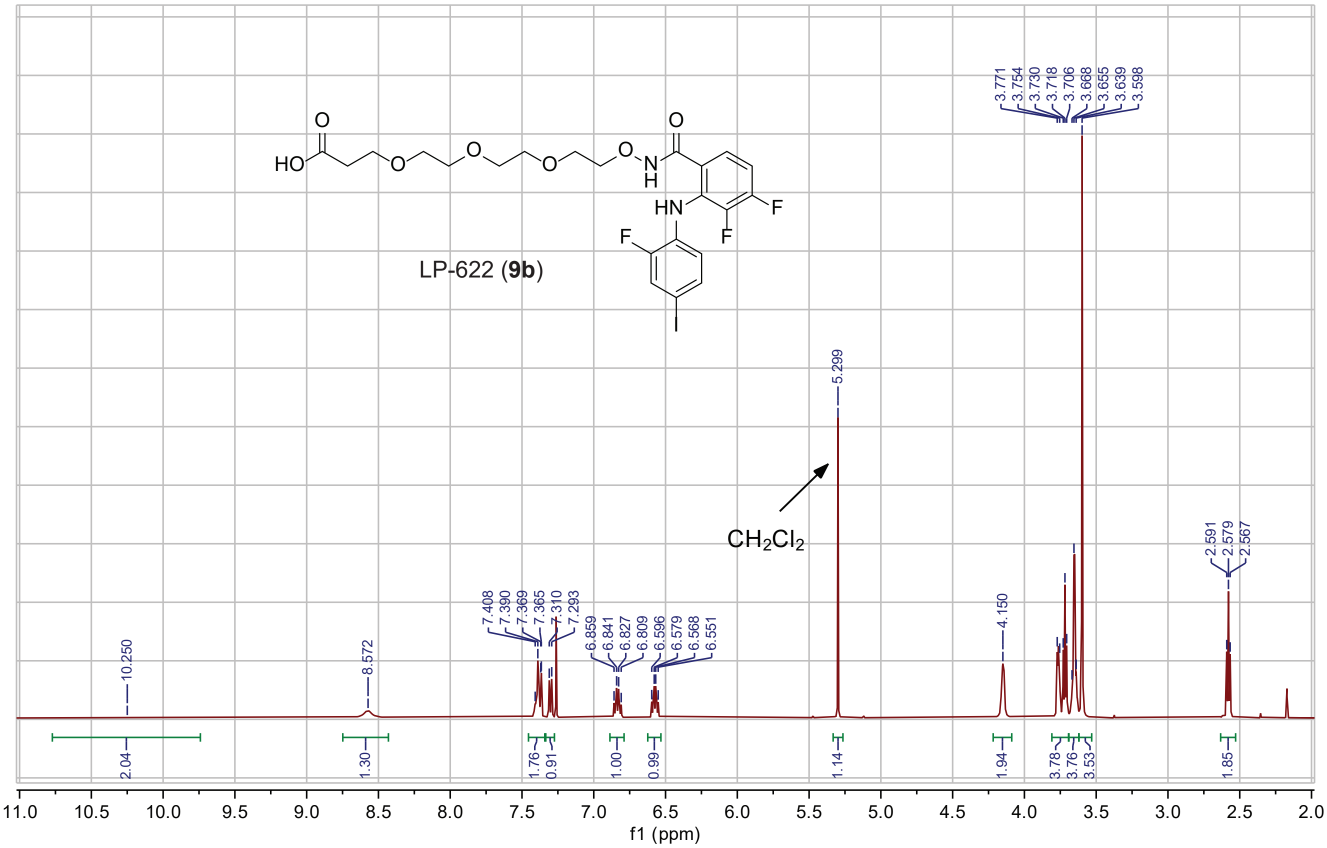

Supplementary Figure 2. **Characterization of key LP-182 synthetic intermediates LP-527 (7) and LP-622 (9b). a** ^{1}H NMR spectra and associated NMR peak assignments for LP-527 (**7**); *N*-(2-chloro-5-(3-(piperazin-1-yl)quinoxalin-6-yl)pyridin-3-yl)-4-fluorobenzenesulfonamide. **b** ^{1}H NMR spectra and associated NMR peak assignments for LP-622 (**9b**); 1-(3,4-difluoro-2-((2-fluoro-4-iodophenyl)amino)phenyl)-1-oxo-3,6,9,12-tetraoxa-2-azapentadecan-15-oic acid.

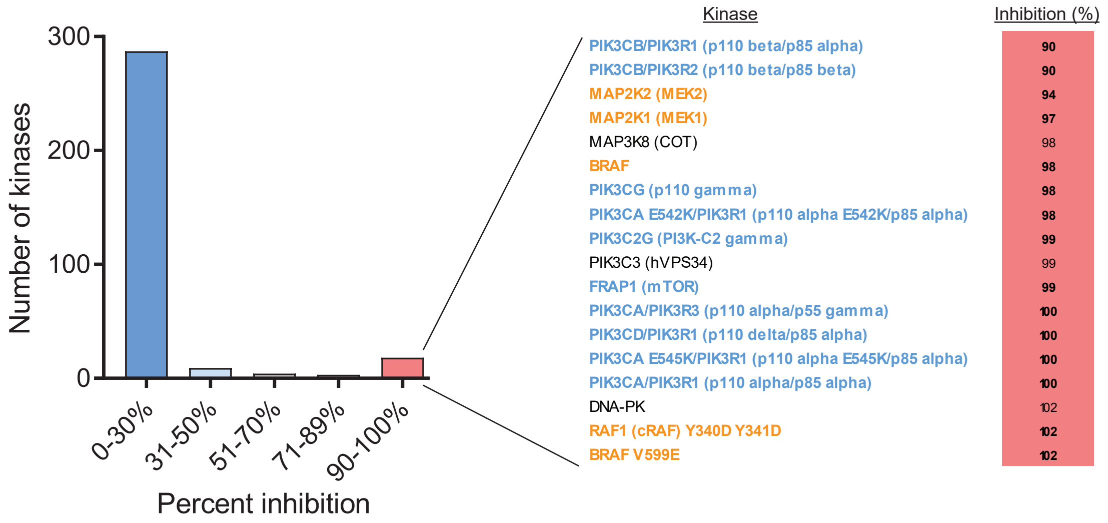

Supplementary Figure 3. **Selectivity and specificity of LP-182 for PI3K, mTOR, RAF, and MEK enzyme targets.** Single-point broad panel kinome screening against 2.5 µM LP-182 (321 kinase isoforms, holoenzymes, or mutant variants). Kinases were grouped based on their average percent inhibition from replicate data as indicated. Color scale follows that used for Coral Human Kinome Visualization analysis and represents percent inhibition as indicated (Fig. 2b). Kinase notation shows the LP-182 targets PI3K/mTOR in blue and RAF/MEK in orange. Average percent kinome inhibition values from replicate data are supplied in Supplementary Data 1.

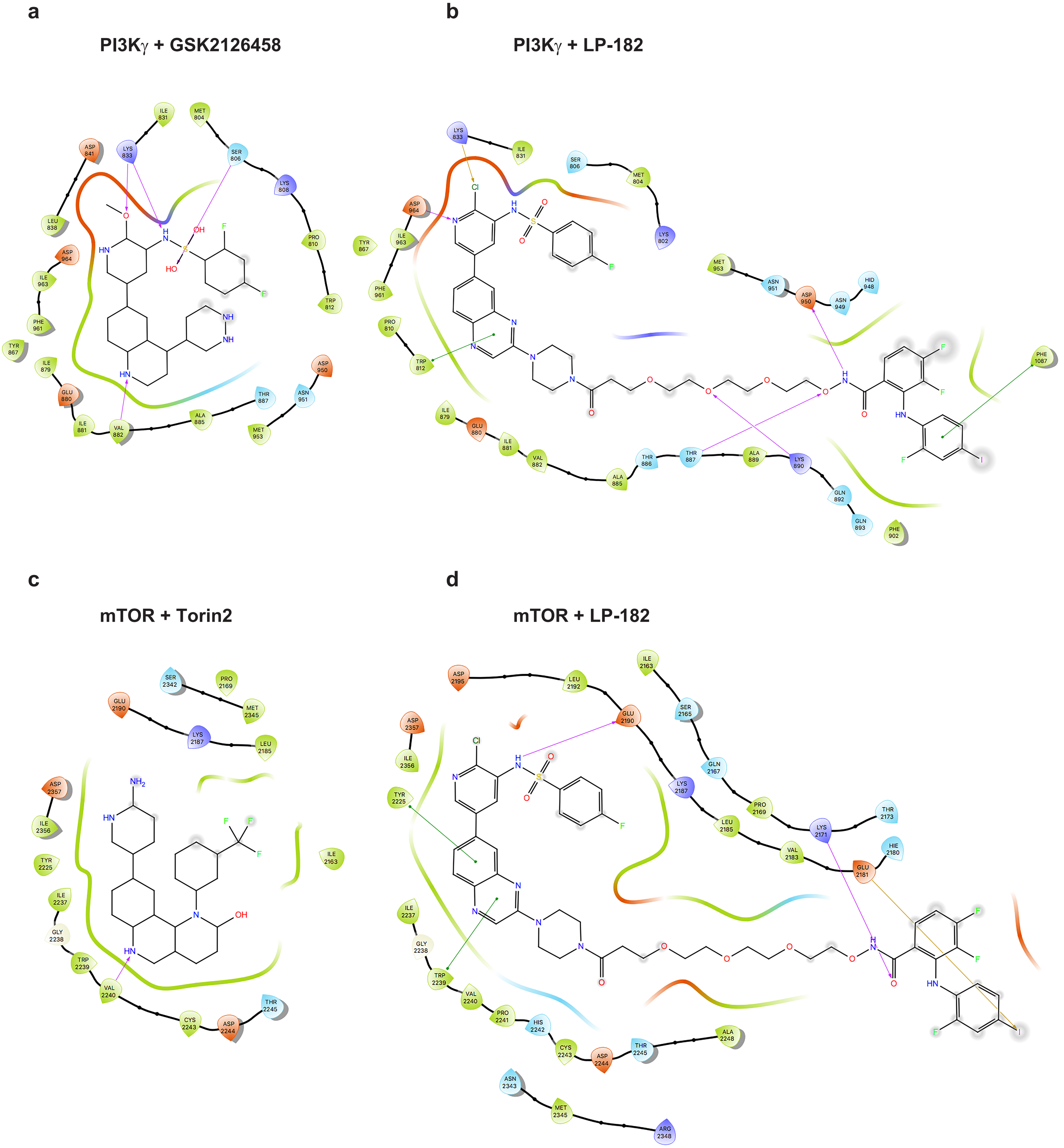

Supplementary Figure 4. **LP-182 maintains binding interactions observed for closely related single agent inhibitor analogs of PI3Kγ and mTOR. a, b** Two-dimensional ligand interaction diagram for PI3Kγ (PDB code 3L08) complexed with **a** GSK2126458[1] or **b** LP-182. **c, d** Two-dimensional ligand interaction diagram for mTOR (PDB code 4JSX) complexed with **c** Torin2[2] or **d** LP-182. Diagrams were generated from the selected 3-D poses using the Schrödinger software tools to highlight protein-ligand interactions. All diagrams represent the best 2-D orientation, rotated to align ligand structures, and cleaned up to normalize orientation and arrangement of groups.

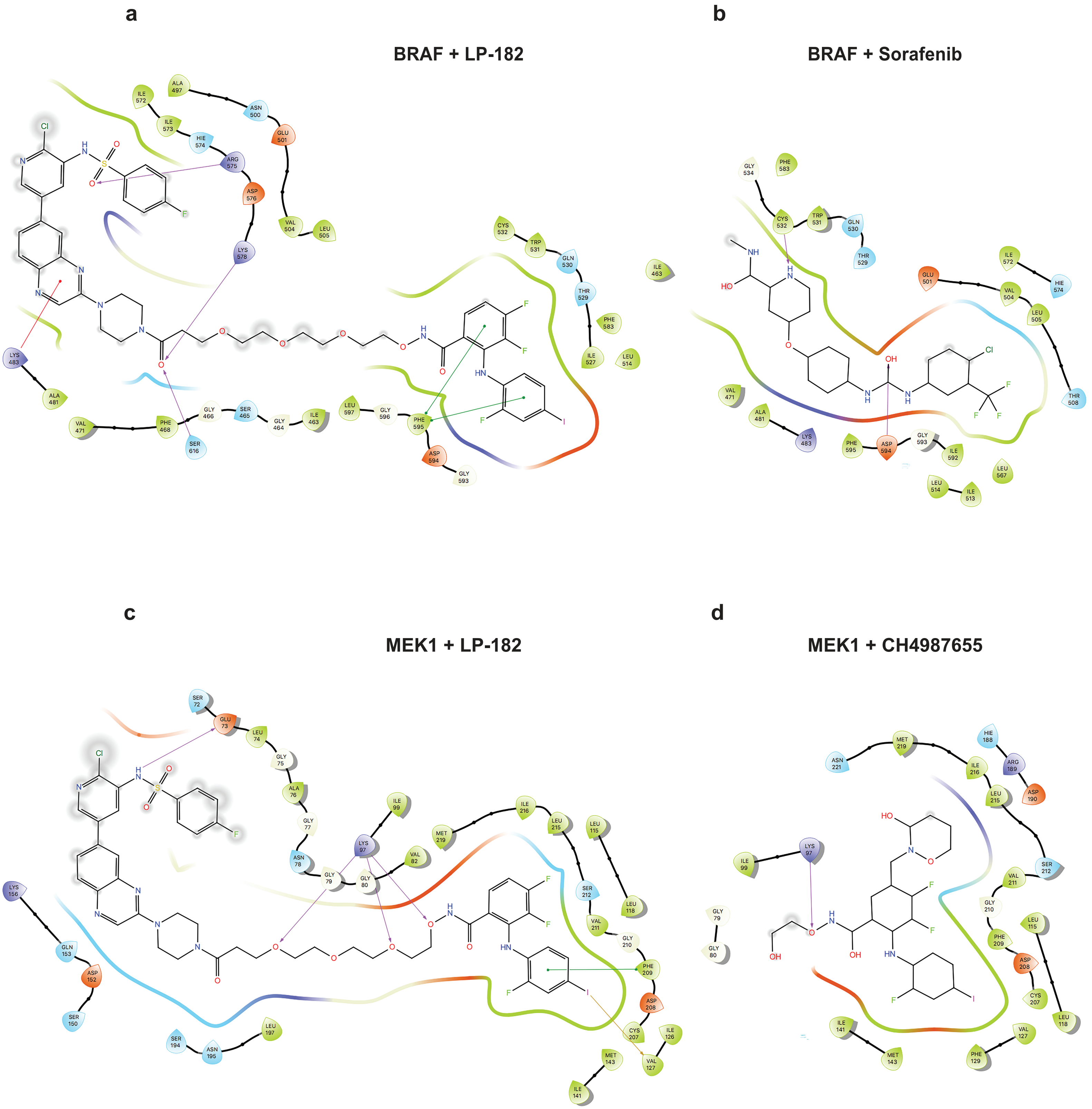

Supplementary Figure 5. **LP-182 maintains binding interactions observed for closely related single agent inhibitor analogs of BRAF and MEK1. a, b** Two-dimensional ligand interaction diagram for BRAF (PDB code 5HI2) complexed with **a** LP-182 or **b** Sorafenib[3]. **c, d** Two-dimensional ligand interaction diagram for MEK1 (PDB code 3ORN) complexed with **c** LP-182 or **d** CH4987655[4]. Diagrams were generated from the selected 3-D poses using the Schrödinger software tools to highlight protein-ligand interactions. All diagrams represent the best 2-D orientation, rotated to align ligand structures, and cleaned up to normalize orientation and arrangement of groups.

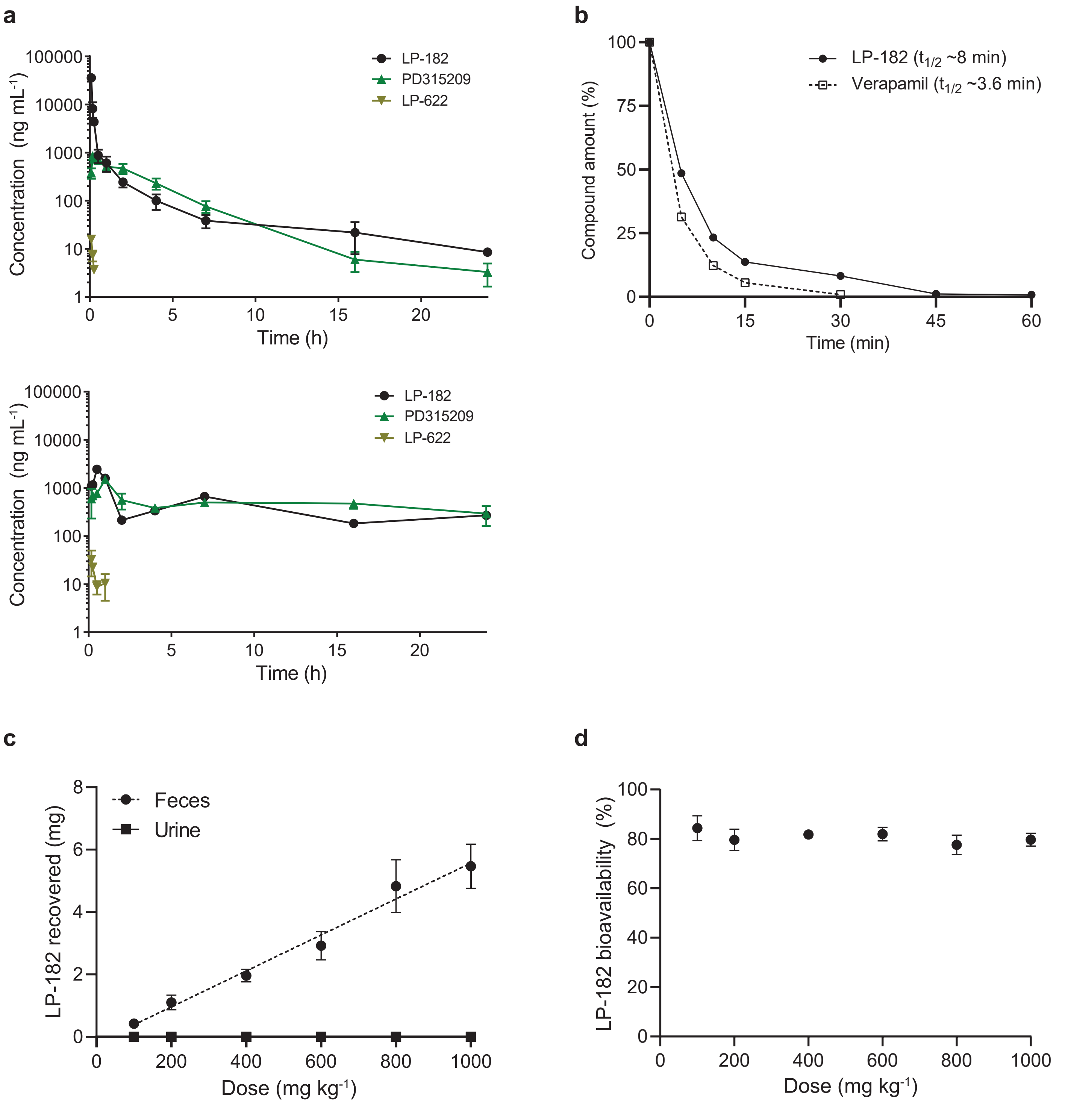

Supplementary Figure 6. **Prolonged dose-independent systemic oral bioavailability of LP-182 and active metabolite inhibitors. a** In vivo pharmacokinetics of LP-182 and associated metabolites in mice following a single i.v. (15 mg kg^{-1}; top panel) or p.o. (400 mg kg^{-1}; bottom panel) dose of LP-182. Data represent the mean ± s.d., n=3 animals sampled per time point per compound dosing. Source data are provided as a Source Data file. **b** Quantitative determination of 1 µM LP-182 or Verapamil (control) stability over 60 min in ~0.5 mg mL^{-1} murine liver microsomes. Data represent the mean, n=1 independent experiment. **c** Cumulative LP-182 recovered from mice feces or urine over 72 h following a single p.o. administration at the indicated doses. Data represents the mean ± s.e.m., n=4 animals per dose. Source data are provided as a Source Data file. **d** Percent oral bioavailability of LP-182 as determined by the percent difference in the amount of compound administered compared to the amount of compound recovered over 72 h of fecal collection following a single p.o. administration at the indicated doses. Data represents the mean ± s.e.m., n=4 animals per dose. LP-182 dose (mg kg^{-1}):LP-182 administered (mg) is 100:2.7; 200:5.4; 400:10.8; 600:16.2; 800:21.6; 1000:27.0. Source data are provided as a Source Data file.

Compound (cLogP)	Chemical structure	mTOR	PI3Kα	PI3Kβ	PI3Kγ	PI3Kδ	BRAF	CRAF	MEK1	MEK2
LP-182 (7.64)	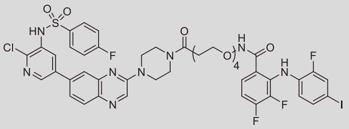	53 ± 2.6	2.2 ± 0.13	342 ± 81	31 ± 2.2	3.6 ± 0.22	49 ± 4.4	79 ± 2.8	104 ± 9.8	150 ± 10.8
LP-527 (7) (3.37)	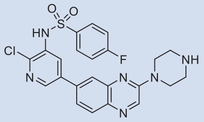	6.1 ± 0.4	0.58 ± 0.05	6.1 ± 0.56	5.0 ± 0.58	0.9 ± 0.05	N/A	N/A	N/A	N/A
GSK2126458 (4.07)	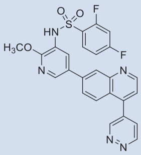	4.2 ± 0.05	0.13 ± 0.004	1.1 ± 0.092	0.8 ± 0.048	0.11 ± 0.005	N/A	N/A	N/A	N/A
PD0316684 (3.68)	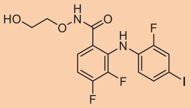	N/A	N/A	N/A	N/A	N/A	6.0 ± 0.52	4.0 ± 0.24	5.7 ± 0.36	18 ± 0.84
PD0325901 (2.85)	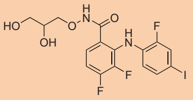	N/A	N/A	N/A	N/A	N/A	6.2 ± 0.29	3.2 ± 0.14	6.5 ± 1.1	17 ± 0.48

Inhibition data (IC_{50}) are shown in nM ± s.e.m.

Supplementary Figure 7. **Target kinase inhibition by LP-182 and active metabolite inhibitors.** Target kinase (PI3K, mTOR, RAF, MEK) 10-point inhibition assays against LP-182, LP-527, GSK2126458, PD0316684, or PD0325901 across concentrations spanning those indicated in Fig. 2c. Data represent the IC_{50} ± s.e.m. (in nM), n ≥ 2 replicates. Corrected data were normalized to vehicle treated control values and analyzed by non-linear regression for determination of IC_{50} values. Source data are provided as a Source Data file.

a

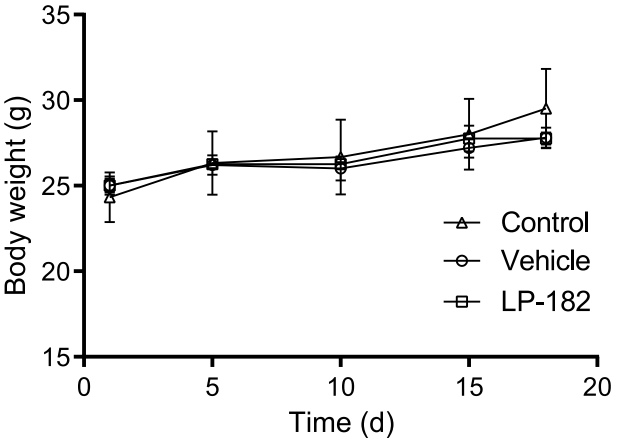

b

Relative organ/body weight (%)	Vehicle	LP-182
Liver	6.051 ± 0.841	5.944 ± 0.467
Left kidney	0.658 ± 0.106	0.661 ± 0.015
Right kidney	0.633 ± 0.071	0.653 ± 0.025
Spleen	0.484 ± 0.147	0.515 ± 0.107
Thymus	0.288 ± 0.033	0.232 ± 0.058
Brain	1.706 ± 0.071	1.672 ± 0.104

Data expressed as mean ± s.d.

c

Hematology analysis	Vehicle	LP-182
White blood cells (10^3 μL^{-1})	9.753 ± 2.773	6.860 ± 2.314
Neutrophils (10^3 μL^{-1})	3.157± 1.499	1.753 ± 0.681
Lymphocytes (10^3 μL^{-1})	5.660 ± 0.707	4.518 ± 1.366
Monocytes (10^3 μL^{-1})	0.473 ± 0.310	0.380 ± 0.369
Eosinophils (10^3 μL^{-1})	0.380 ± 0.529	0.178 ± 0.056
Basophils (10^3 μL^{-1})	0.083 ± 0.104	0.038 ± 0.017
Red blood cells (10^6 μL^{-1})	8.243 ± 0.499	9.040 ± 0.438
Hemoglobin (g dL^{-1})	12.930 ± 0.971	14.180 ± 0.591
Hematocrit (%)	46.530 ± 2.386	49.450 ± 1.838
MCV (fL)	56.470 ± 0.586	54.800 ± 3.354
MCH (pg)	15.700 ± 0.265	15.700 ± 0.770
MCHC (g dL^{-1})	27.770 ± 0.702	28.650 ± 0.666
RDW (%)	15.270 ± 0.635	16.430 ± 0.896
Platelets (10^3 μL^{-1})	758.3 ± 91.24	620.8 ± 405.7
Mean platelet volume (fL)	4.833 ± 0.643	5.300 ± 0.530

Data expressed as mean ± s.d.

MCV, mean corpuscular volume; MCH, mean corpuscular hemoglobin; MCHC, mean corpuscular hemoglobin concentration; RDW, red cell distribution width

d

Histological findings	Vehicle	LP-182
Eyes	0 of 10	0 of 8
Heart	0 of 5	0 of 4
Lungs	0 of 5	0 of 4
Liver	0 of 5	0 of 4
Kidneys	0 of 10	0 of 8
Thymus	0 of 5	0 of 4
Stomach	0 of 5	0 of 4
Duodenum	0 of 5	0 of 4
Jejunum	0 of 5	0 of 4
Ileum	0 of 5	0 of 4
Pancreas	0 of 5	0 of 4
Cecum	0 of 5	0 of 4
Colon	0 of 5	0 of 4
Skin	0 of 5	0 of 4
Spleen	0 of 5	0 of 4
Thymus	0 of 5	0 of 4
Mesenteric lymph nodes	0 of 5	0 of 4
Bone marrow (sternal)	0 of 5	0 of 4

Supplementary Figure 8. **Daily treatment with LP-182 exhibits no observed toxicity *in vivo*.** **a** Body weight of untreated control mice or of mice following daily oral administration of vehicle or LP-182 at 400 mg kg^{-1} for 10 d followed by 8 d rest. Data represent the mean ± s.e.m., n=3 (untreated control), n=5 (vehicle), n=4 (LP-182) animals. Statistical significance determined using multiple unpaired t-test corrected with Holm-Šidák multiple comparisons test. No significant differences determined. Source data are provided as a Source Data file. **b** Relative percent organ to body weight of mice following daily oral administration of vehicle or LP-182 at 400 mg kg^{-1} for 10 d followed by 8 d rest. Data represent the mean ± s.d., n=5 (vehicle), n=4 (LP-182) animals. Statistical significance determined using two-tailed unpaired t-test. No significant differences determined. Source data are provided as a Source Data file. **c** Hematological analysis of mice following daily oral administration of vehicle or LP-182 at 400 mg kg^{-1} for 10 d followed by 8 d rest. Data represent the mean ± s.d., n=3 (vehicle), n=4 (LP-182) animals. Statistical significance determined using two-tailed unpaired t-test. No significant differences determined. Source data are provided as a Source Data file. **d** Histological observations in mice following daily oral administration of vehicle or LP-182 at 400 mg kg^{-1} for 10 d followed by 8 d rest. Data represent number of adverse findings out of total organs/tissues analyzed, n=5 (vehicle), n=4 (LP-182) animals.

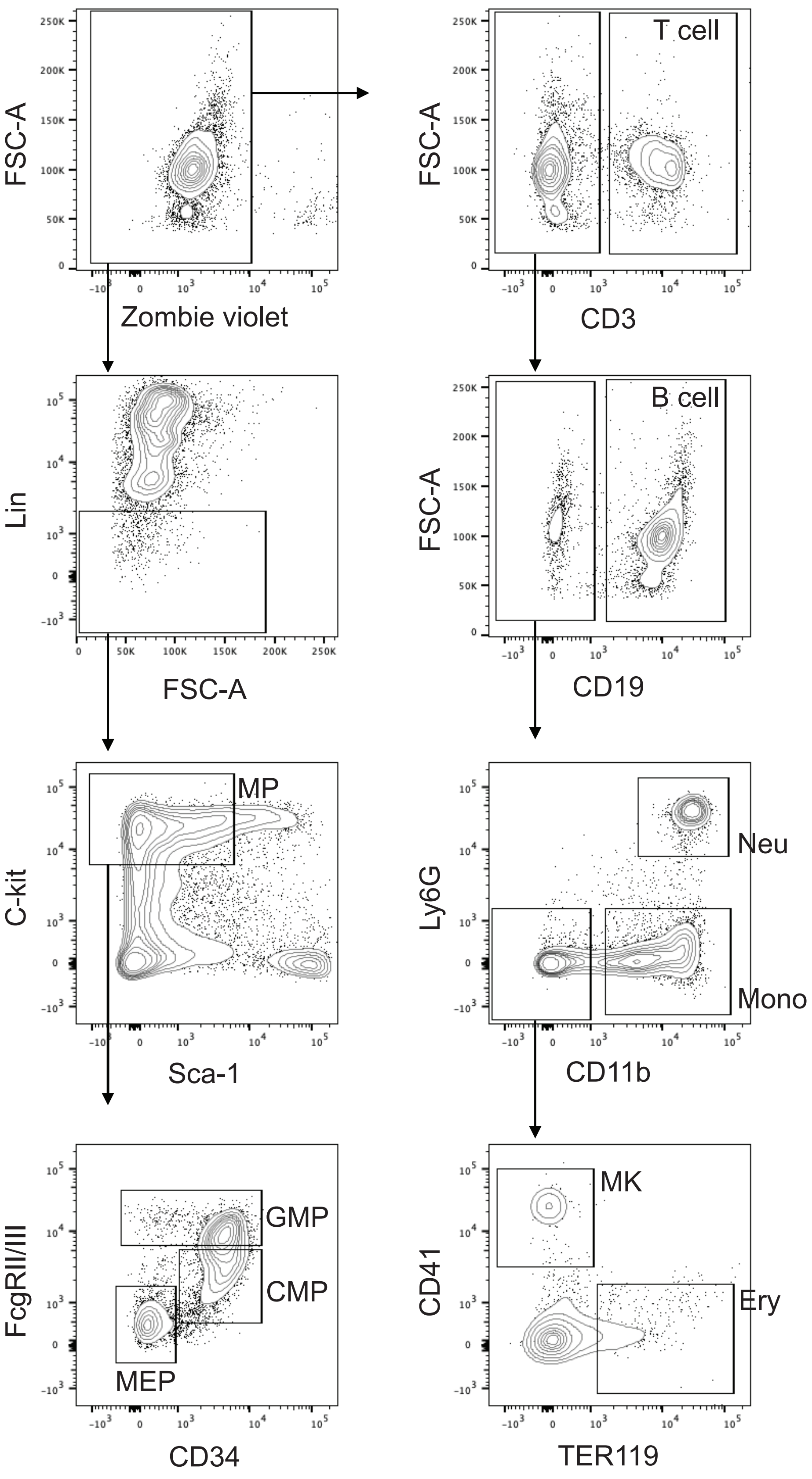

Supplementary Figure 9. **Gating strategy for flow cytometry.** Using the FSC/SSC gating, debris was removed by gating on the main cell population. Using the FSC-W/FSC-H and SSC-W/SSC-H gating, singlets were selected. Positivity threshold for each parameter was defined on the basis of mock-treated (DMSO) sample or isotype control staining. Identical positivity threshold was applied to all samples within cell line and tissue.

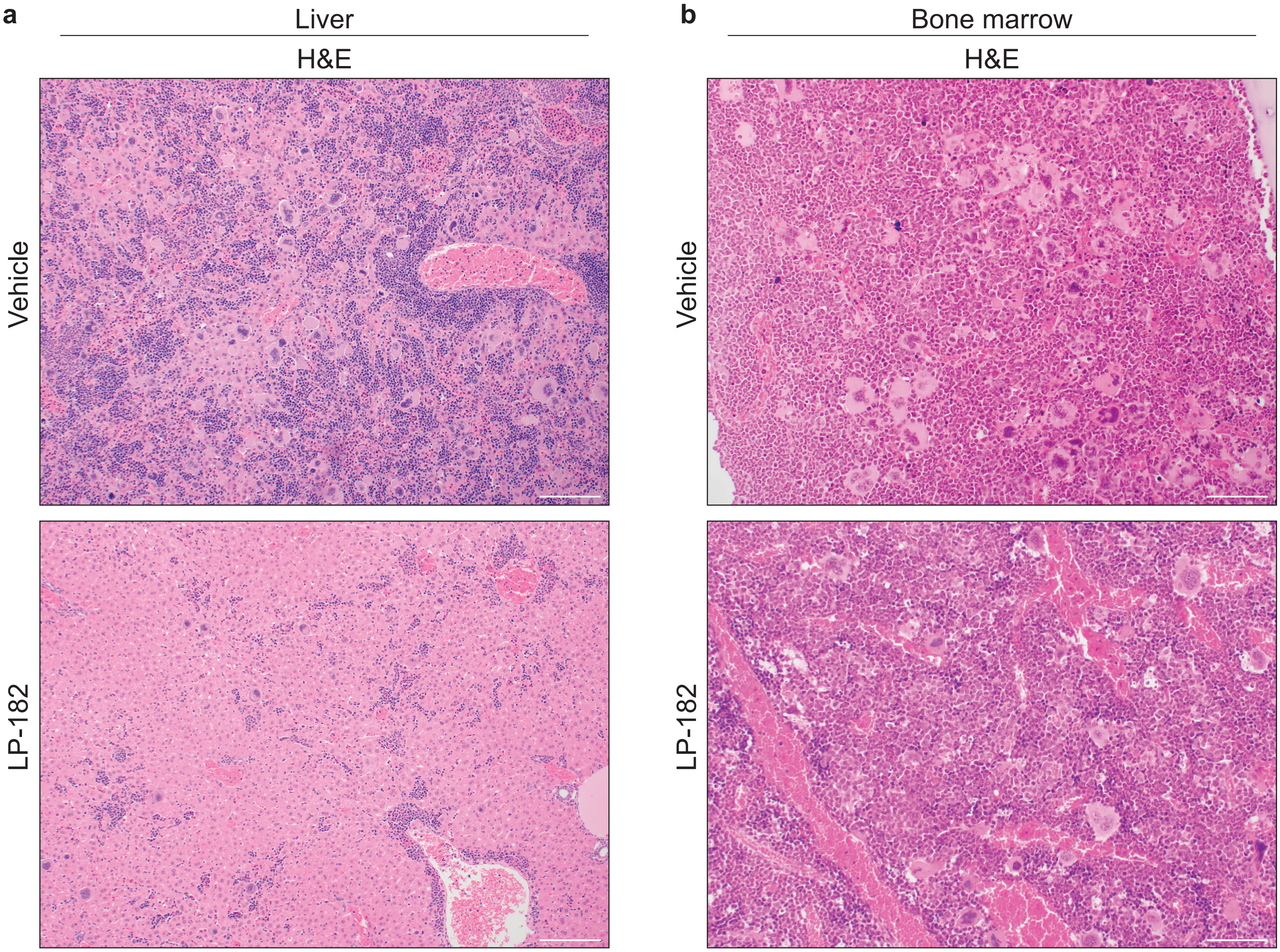

Supplementary Figure 10. **Histopathology changes in cellularity by LP-182 treatment in the *MPL*^{W515L} mouse model of myelofibrosis. a** Representative histological images from liver of myelofibrosis mice treated as indicated. Hematoxylin & Eosin (H&E; 10x, scale 100 μm). Data representative of vehicle (n = 4), LP-182 (n = 5). **b** Representative histological images from bone marrow of myelofibrosis mice treated as indicated. H&E (20x, scale 50 μm). Data representative of vehicle (n = 4), LP-182 (n = 5).

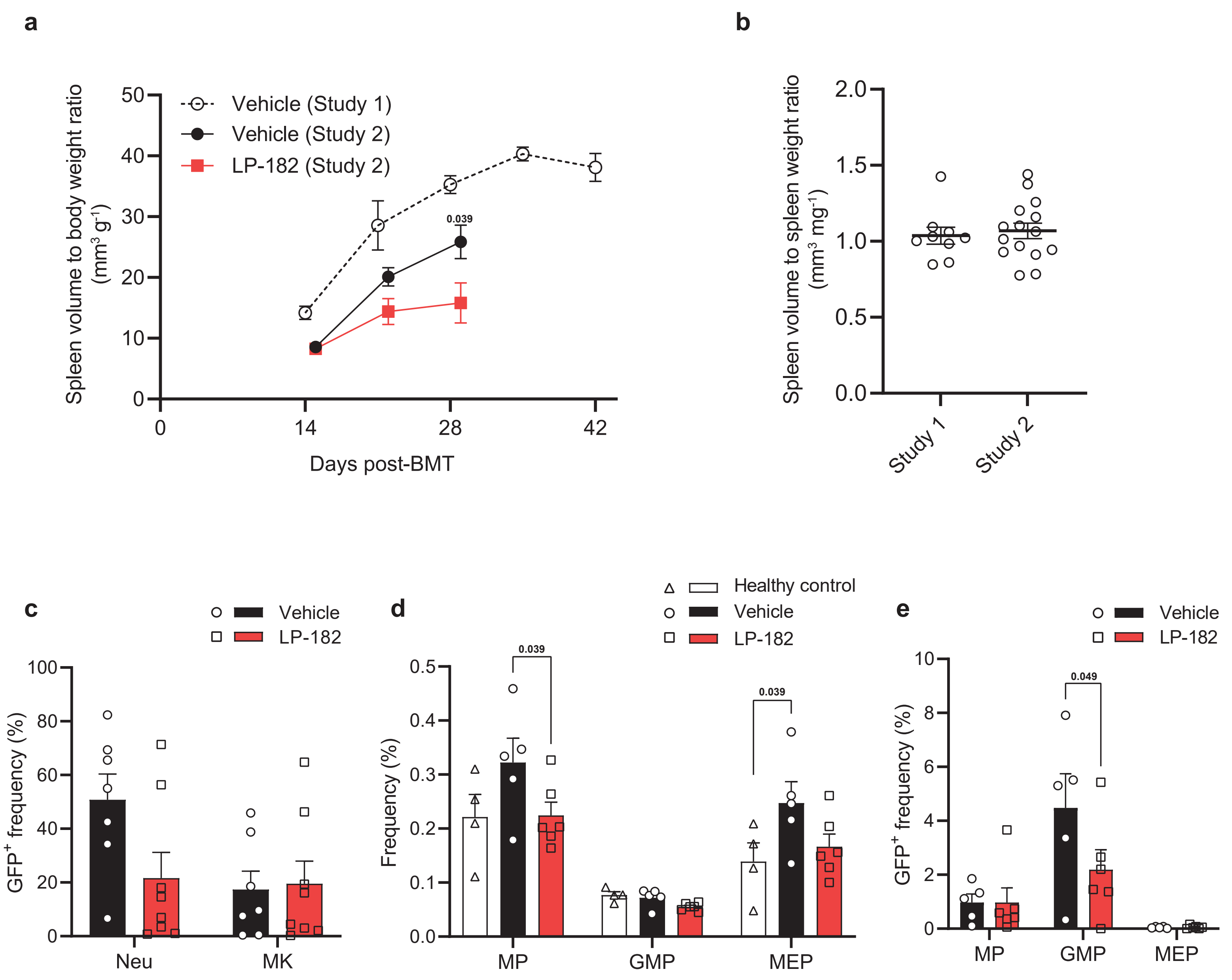

Supplementary Figure 11. **Volumetric magnetic resonance imaging and flow cytometry display changes in cellularity, and accurately and consistently reflect splenomegaly reduction by LP-182 in the *MPL*^{W515L} mouse model of myelofibrosis. a** Spleen volume to body weight measurements of vehicle or LP-182 treated animals on the indicated day post-bone marrow transplant (BMT). Data represent the mean ± s.e.m., n=4 (vehicle; Study 1), n=7 (vehicle; Study 2), n=8 (LP-182; Study 2). Statistical significance for Study 2 values determined using two-tailed unpaired t-test (Study 2 Vehicle vs. LP-182 at 28 d, p = 0.039). Source data are provided as a Source Data file. **b** Combined magnetic resonance spleen volume to gravimetric spleen weight measurements from vehicle and LP-182 treated animals on day of tissue harvest. Data represent the mean ± s.e.m., n=9 (Study 1), n=15 (Study 2). Details regarding individual studies are supplied in the Results and Methods sections of the manuscript. **c-e** Frequency of **c** GFP-positive Neu and MK cells, **d** total, and **e** GFP-positive progenitor cells from spleen of healthy control and *MPL*^{W515L} MF mice treated p.o. daily for 14 d with vehicle or LP-182 at 400 mg kg^{-1}. Data represent the mean ± s.e.m, healthy control (n = 4), vehicle (n = 7, **c**; n = 5, **d**; n = 5, **e**), LP-182 (n = 8, **c**; n = 6, **d**; n = 6, **e**) animals per group. Statistical significance determined using Two-way ANOVA corrected with either Šidák's (**c**, **e**) or Tukey's (**d**) multiple comparisons test (MP Vehicle vs. LP-182, p = 0.039; MEP Healthy control vs. Vehicle, p = 0.039; GMP Vehicle vs. LP-182, p = 0.049). Green-fluorescent protein, GFP; Megakaryocyte, MK; Neutrophil, Neu; Myeloid progenitor, MP; granulocyte-monocyte progenitor, GMP; Megakaryocyte-erythrocyte progenitor, MEP.

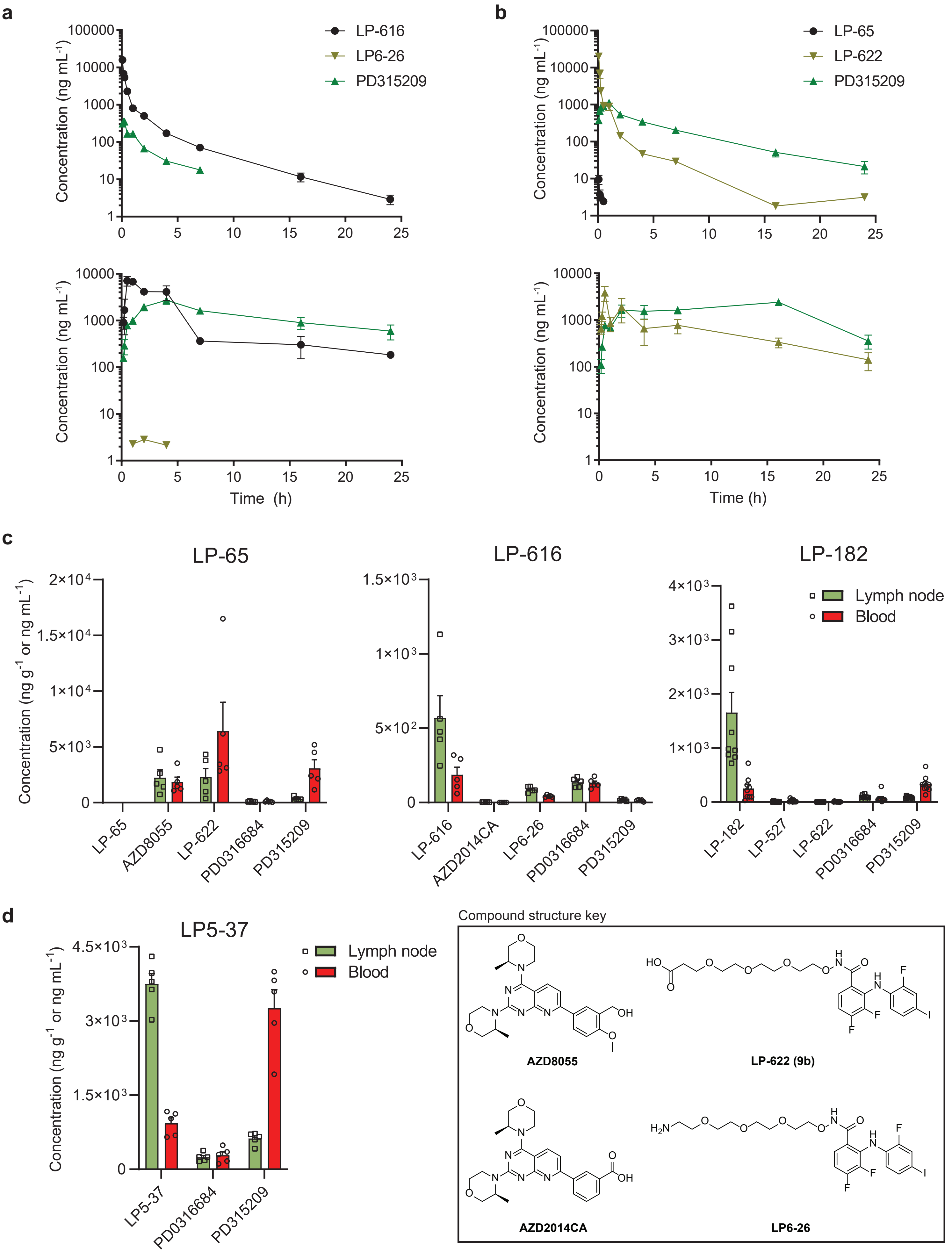

a
LP-616
LP6-26
PD315209
Concentration (ng mL-1)
Time (h)
b
LP-65
LP-622
PD315209
Concentration (ng mL-1)
Time (h)
c
LP-65
LP-616
LP-182
Concentration (ng g-1 or ng mL-1)
Lymph node
Blood
LP-65
AZD8055
LP-622
PD0316684
PD315209
LP-616
AZD2014CA
LP6-26
LP-182
LP-527
d
LP5-37
Concentration (ng g-1 or ng mL-1)
Lymph node
Blood
PD0316684
PD315209
Compound structure key
AZD8055
LP-622 (9b)
AZD2014CA
LP6-26

Supplementary Figure 12. **Physiochemical properties influence lymphatic uptake and metabolite distribution of lymphatropic kinase inhibitors. a, b** *In vivo* pharmacokinetics of **a** LP-616 or **b** LP-65 and associated metabolites in mice following a single intravenous (15 mg kg^{-1}; top panel) or oral (200 mg kg^{-1}; bottom panel) dose of LP-616 or LP-65. Data represent the mean ± s.e.m., LP-616 (n = 6, 10 and 60 min timepoints, oral; n = 3, all other timepoints, intravenous and oral), LP-65 (n = 3, all timepoints, intravenous and oral) animals per group. Source data are provided as a Source Data file. **c** Concentration of LP-65, LP-616, LP-182, and associated metabolites in blood and mesenteric lymph nodes from mice 4 h following oral administration at 400 mg kg^{-1}. Data represents the mean ± s.e.m., LP-65 and LP-616 (n = 5), LP-182 (n = 9) animals per group. **d** Concentration of LP5-37 and associated metabolites in blood and mesenteric lymph nodes from mice 4 h following oral administration at 400 mg kg^{-1}. Data represents the mean ± s.e.m., n = 5.

Scoring	
**Megakaryocyte/High-power field**	Average of 5 High-power fields at 40x
**Effacement of spleen architecture**	Subjective assessment based on the extent of extramedullary hematopoiesis and loss of white pulp
**Liver extramedullary hematopoiesis**	Subjective assessement of the extent of extramedullary hematopoiesis and distortion of liver architectures
**Atypical megakaryocytes**	Large hyperlobulated megakaryocytes with open chromatin and variably prominent nucleoli
**Large megakaryocyte clustering**	Tight clusters with > 7 megakaryocytes
**GFP-positive Megakaryocyte/High-power field**	Average of 5 High-power fields at 40x. Clusters prioritized
**Reticulin fibrosis**	WHO grading system

**Spleen**						**Liver**
**Cohort**	**Treatment**	**Megakaryocyte/High-power field**	**Granz Hyperplasia**	**Effacement of architecture**		**Extramedullary hematopoiesis**
Study 1	Vehicle	26	Large cohesive sheets	Marked		Severe
Study 1	Vehicle	38.8	Large cohesive sheets	Marked		Severe
Study 1	Vehicle	46.4	Large cohesive sheets	Marked		Severe
Study 1	Vehicle	39.6	Large cohesive sheets	Marked		Severe
Study 1	LP-182	13.4	Small aggregates	Mild		Mild
Study 1	LP-182	4.2	Not present	None		Mild
Study 1	LP-182	12.4	Small aggregates	Moderate		Moderate
Study 1	LP-182	20	Moderate aggregates	Moderate		Severe
Study 1	LP-182	32.8	Large cohesive sheets	Marked		Severe

**Spleen**					
**Cohort**	**Treatment**	**GFP-positive Megakaryocyte/High-power field**	**GFP-negative Megakaryocyte/High-power field**	**Average total Megakaryocyte/ High-power field**	**GFP-positive Megakaryocyte Frequency**
Study 1	Vehicle	32.2	2.8	35	92.0
Study 1	Vehicle	37.8	5	42.8	88.3
Study 1	Vehicle	43.8	3.2	47	93.2
Study 1	Vehicle	40.8	2.6	43.4	94.0
Study 1	LP-182	19.8	4.2	24	82.5
Study 1	LP-182	15.8	4.2	20	79.0
Study 1	LP-182	20.2	4	24.2	83.5
Study 1	LP-182	30.4	3.8	34.2	88.9

Note: Intensity of stain not always correlative with atypia.
GFP-positive myeloid cells also present.
Staining variable among sections and regions of the spleen (edge more consistent).

**Bone Marrow (Femur)**						
**Cohort**	**Treatment**	**Cellularity**	**Megakaryocyte/High-power field**	**Atypical Megakaryocytes**	**Large Megakaryocyte clustering**	**Reticulin fibrosis**
Study 1	Vehicle	>95%	18.6	Many	Present	2
Study 1	Vehicle	>95%	36.2	Many	Present	2
Study 1	Vehicle	>95%	26.4	Many	Present	2
Study 1	Vehicle	>95%	34.8	Many	Present	2
Study 1	LP-182	>95%	13.2	Many	Absent	0
Study 1	LP-182	90%	9.4	None	Absent	0
Study 1	LP-182	>95%	19.2	Many	Present	1
Study 1	LP-182	>95%	43	Many	Present	2
Study 1	LP-182	>95%	41.6	Many	Present	1

Supplementary Table 1. **Histological assessment and scoring morphologic hallmarks of MF shows less severe progression upon treatment with LP-182.** Scoring metrics, parameters, and individual data sets for quantitation of megakaryocytes from an average of 5 high-power fields (40x), WHO grading of reticulin fibrosis in bone marrow of the femur[5, 6], effacement of spleen architecture, and assessment of spleen and liver extramedullary hematopoiesis (EMH).

Supplementary references

1. Knight SD*, et al.* Discovery of GSK2126458, a Highly Potent Inhibitor of PI3K and the Mammalian Target of Rapamycin. *ACS Med Chem Lett* **1**, 39-43 (2010).

2. Yang H, Rudge DG, Koos JD, Vaidialingam B, Yang HJ, Pavletich NP. mTOR kinase structure, mechanism and regulation. *Nature* **497**, 217-223 (2013).

3. Foster SA*, et al.* Activation Mechanism of Oncogenic Deletion Mutations in BRAF, EGFR, and HER2. *Cancer Cell* **29**, 477-493 (2016).

4. Isshiki Y*, et al.* Design and synthesis of novel allosteric MEK inhibitor CH4987655 as an orally available anticancer agent. *Bioorg Med Chem Lett* **21**, 1795-1801 (2011).

5. Thiele J, Kvasnicka HM, Facchetti F, Franco V, van der Walt J, Orazi A. European consensus on grading bone marrow fibrosis and assessment of cellularity. *Haematologica* **90**, 1128-1132 (2005).

6. Swerdlow SH*, et al.* WHO Classification of Tumours of Haematopoietic and Lymphoid Tissues.). 4th edn. International Agency for Research on Cancer (4th edn., 2017).
